# Inhibition of exchange proteins directly activated by cAMP as a strategy for broad-spectrum antiviral development

**DOI:** 10.1016/j.jbc.2023.104749

**Published:** 2023-04-24

**Authors:** Stephen Boulton, Mathieu J.F. Crupi, Siddharth Singh, Madalina E. Carter-Timofte, Taha Azad, Bailey C. Organ, Xiaohong He, Rida Gill, Serge Neault, Taylor Jamieson, Jaahnavi Dave, Naziia Kurmasheva, Bradley Austin, Julia Petryk, Ragunath Singaravelu, Ben Zhen Huang, Noah Franco, Kaaviya Babu, Robin J. Parks, Carolina S. Ilkow, David Olagnier, John C. Bell

**Affiliations:** 1 Cancer Therapeutics Program, Ottawa Hospital Research Institute, Ottawa, Ontario, Canada; 2 Department of Biochemistry, Microbiology and Immunology, University of Ottawa, Ottawa, Ontario, Canada; 3 Aarhus University, Department of Biomedicine, Aarhus C, Denmark; 4 Faculty of Medicine and Health Sciences, Department of Microbiology and Infectious Diseases, Université de Sherbrooke, Sherbrooke, Canada; 5 Centre de Recherche Du CHUS, Sherbrooke, Canada; 6 Public Health Agency of Canada, Ottawa, Ontario, Canada; 7 Department of Medicine, University of Ottawa, Ottawa, Ontario, Canada

**Keywords:** actin, adenovirus, antiviral, COVID-19, EPAC, ESI-09, HSV-1, measles, mpox, Rho GTPases, SARS-CoV-2, VSV, vaccinia

## Abstract

The recent SARS-CoV-2 and mpox outbreaks have highlighted the need to expand our arsenal of broad-spectrum antiviral agents for future pandemic preparedness. Host-directed antivirals are an important tool to accomplish this as they typically offer protection against a broader range of viruses than direct-acting antivirals and have a lower susceptibility to viral mutations that cause drug resistance. In this study, we investigate the exchange protein activated by cAMP (EPAC) as a target for broad-spectrum antiviral therapy. We find that the EPAC-selective inhibitor, ESI-09, provides robust protection against a variety of viruses, including SARS-CoV-2 and Vaccinia (VACV)—an orthopox virus from the same family as mpox. We show, using a series of immunofluorescence experiments, that ESI-09 remodels the actin cytoskeleton through Rac1/Cdc42 GTPases and the Arp2/3 complex, impairing internalization of viruses that use clathrin-mediated endocytosis (*e.g.* VSV) or micropinocytosis (*e.g.* VACV). Additionally, we find that ESI-09 disrupts syncytia formation and inhibits cell-to-cell transmission of viruses such as measles and VACV. When administered to immune-deficient mice in an intranasal challenge model, ESI-09 protects mice from lethal doses of VACV and prevents formation of pox lesions. Altogether, our finding shows that EPAC antagonists such as ESI-09 are promising candidates for broad-spectrum antiviral therapy that can aid in the fight against ongoing and future viral outbreaks.

The recent outbreaks with SARS-CoV-2 and mpox (formerly known as monkeypox) have highlighted the global threat that viral infections have on public health. Over the past 50 years, only 90 antiviral drugs, targeting only nine human infectious diseases, have received FDA approval (1). The majority of these drugs are direct-acting antivirals (DAAs), which target specific viral proteins (1, 2). DAAs have good efficacy and a low risk of toxicity but are also often limited in scope against different species (and in some cases, different strains) of viruses which exhibit high genetic diversity (1, 2, 3). In addition, DAAs impose a selective pressure against their targets that increases the possibility of developing resistance against them (2, 3). DAAs also have a lengthy development process, which limits their ability for usage against emerging viral outbreaks. This was one of the major challenges during the COVID-19 pandemic. Despite two prior coronavirus outbreaks within the past 20 years, no antivirals against coronaviruses were available at the onset of the COVID-19 pandemic. This in part stemmed from a lengthy development process in combination with relatively short-lived outbreaks that eventually resolved themselves.

One solution to develop drugs against emerging viruses is by repurposing already approved broad-spectrum antivirals. In the early stages of the COVID-19 pandemic, screening with libraries of broad spectrum antivirals identified remdesivir and molnupiravir, two nucleoside analogs, as promising candidates for COVID-19 treatment (4, 5, 6, 7). Both drugs were used throughout the pandemic to manage symptoms and improve outlook. However, mutations have already developed in the RNA-dependent RNA polymerase of SARS-CoV-2 that confer resistance to remdesivir (8, 9), and similar issues have also been observed with nucleoside analogs in other viruses, in part due to targeting viral proteins that are prone to mutations (10, 11, 12, 13). In contrast to DAAs, host-targeting antivirals target host genes that are hijacked by viruses during different stages of their replication cycle. Since therapies are targeted against the host, there is a higher risk of toxicity, but since many viruses use similar host pathways for replication, they often have broad-spectrum activity against many viruses (2). They are also much less prone to mutations, so there is a higher barrier to overcome for developing drug resistance.

Following the SARS-CoV-2 and mpox outbreaks, there is a pressing need to establish a larger catalog of broad-spectrum antivirals for increased “off-the-shelf” medical countermeasures to aid in future pandemic preparedness and responsiveness. An interesting target for broad-spectrum antiviral therapy is the exchange protein activated by cAMP (EPAC). EPAC is a signaling protein that functions as a guanine exchange factor for RAS-like GTPases and is involved in various pathways regulating cellular adhesion, matrix adhesion, and migration, as well as processes involving vesicle trafficking, calcium homeostasis, cytoskeleton remodeling, and gene expression (14, 15). Many pathways regulated by EPAC are also exploited by viruses for replication. For instance, actin filaments, which are regulated by EPAC *via* Rho family GTPases (14, 16), are involved in attachment, endocytosis, fusion, assembly, and egress for many different viruses (17).

ESI-09 (α-[(2-(3-Chlorophenyl)hydrazinylidene]-5-(1,1-dimethylethyl)-β-oxo-3-isoxazolepropane-nitrile) (Fig. 1*A*) is an EPAC-specific inhibitor that competitively displaces cAMP from the regulatory cAMP-binding domain and allosterically blocks guanine exchange activity (18, 19, 20). It inhibits both isoforms of EPAC with IC_50_ values in the low micromolar range and exhibits selectivity towards EPAC that is more than 100 fold higher than other cAMP receptors such as protein kinase A (20). It has also been shown previously that ESI-09 prevents SARS-CoV-1 and MERS-CoV infection (21). Although, its antiviral activity has not been tested against SARS-CoV-2.

**Figure 1:**
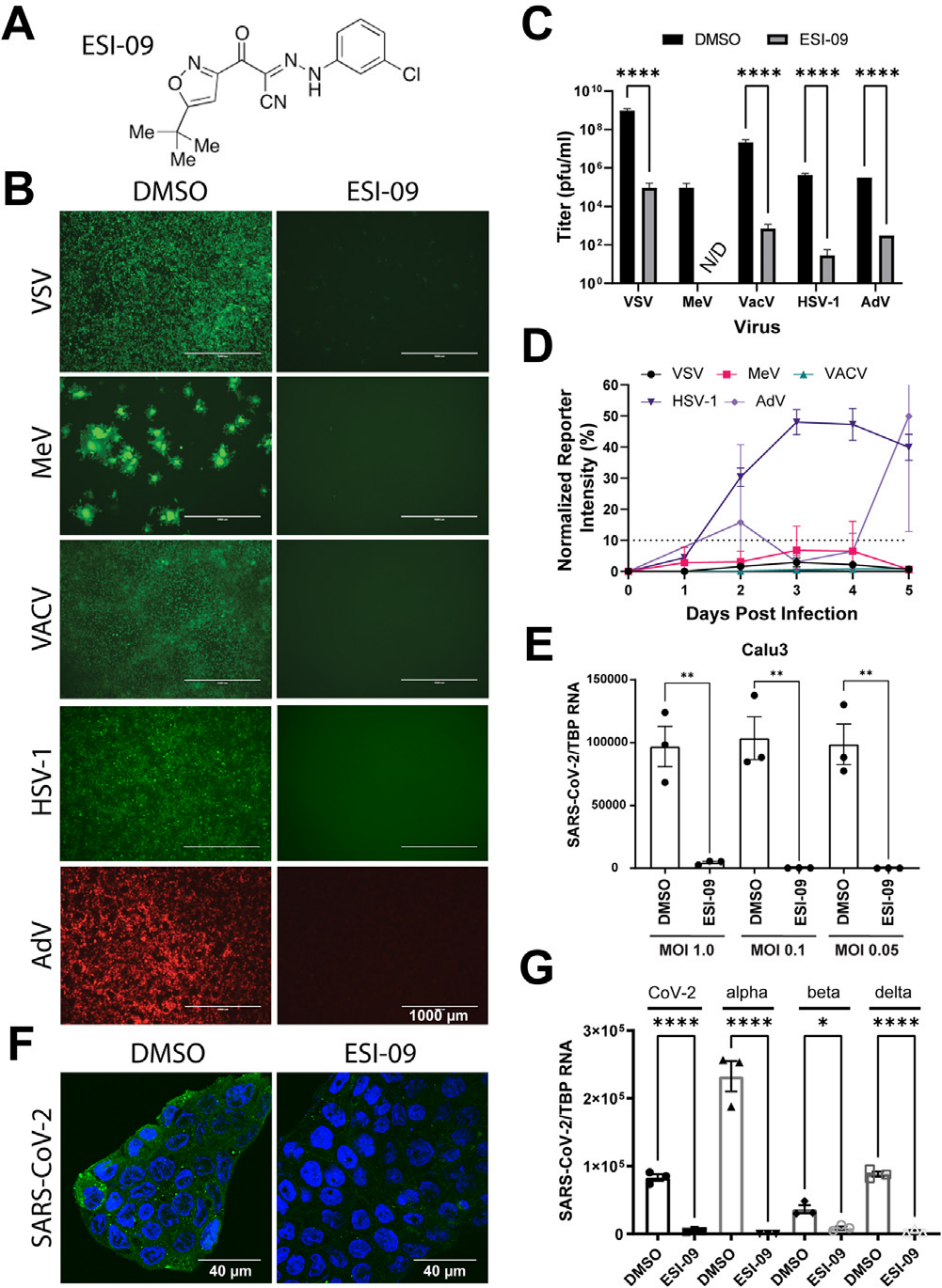
**ESI-09 exhibits antiviral activity against multiple viruses.***A*, Chemical structure of ESI-09. *B*, fluorescence images of U2OS (VACV), Vero (MeV, VSV, and HSV-1), and 293T (AdV) cells treated with DMSO or ESI-09 at a concentration of 25 μM and infected with viruses expressing eGFP or mCherry. Images taken 24 h after infection. *C*, measurement of infectious virus titers from infected cells described in panel A. N/D indicates no infectious virus particles were detectable. *D*, kinetic response of ESI-09 antiviral activity in Vero cells infected with viruses at an MOI of 0.1. Virus replication was measured *via* reporter signal in ESI-09–treated cells (25 μM) normalized to DMSO-treated cells. *E*, immunostaining of SARS-CoV-2 spike protein in Calu3 cells treated with 25 μM ESI-09 or DMSO and infected with SARS-CoV-2 (MOI 0.1). *F*, quantification of SARS-CoV-2 RNA levels in Calu3 cells treated with ESI-09 prior to infection, normalized to TATA-binding protein (TBP) RNA. *G*, quantification of viral RNA levels for SARS-CoV-2 and several of its variants of concern in Vero-E6 hTMPRSS2 cells pretreated with ESI-09 before infection (MOI 0.1). Viral RNA levels were determined 48 h post infection by RT-qPCR. VACV, Vaccinia; VSV, vesicular stomatitis virus; MeV, measles virus; HSV, herpes simplex virus-1; AdV, adenovirus; MOI, multiplicity of infection. Statistical significance determined by Fisher's LSD test (∗*p* < 0.0361; ∗∗*p* < 0.0021; ∗∗∗*p* < 0.0002; ∗∗∗∗*p* < 0.0001).

In this study, we investigate the potential of EPAC as a broad-spectrum antiviral target using its inhibitor, ESI-09, against a small panel of RNA and DNA viruses that include both enveloped and nonenveloped viruses. We find that ESI-09 exhibits antiviral activity against viruses from a variety of families, which include SARS-CoV-2, the etiological agent behind the COVID-19 pandemic, and Vaccinia (VACV), an orthopoxvirus from the same family as mpox. In addition, we investigate the antiviral mechanism of action of ESI-09, revealing that it impairs various stages of the virus life cycle by remodeling parts of the host cytoskeleton leading to inhibition of viral entry and cell-to-cell transmission. Lastly, we demonstrate the therapeutic antiviral potential of ESI-09 against VACV infection in a combination of *ex vivo* and *in vivo* human and murine models.

## Results

### The EPAC-selective inhibitor ESI-09 has broad-spectrum antiviral activity

The antiviral activity of ESI-09 was assayed against five different viruses including both DNA and RNA viruses as well as enveloped and nonenveloped viruses (vesicular stomatitis virus (VSV), measles virus (MeV), VACV virus, herpes simplex virus 1 (HSV-1), and adenovirus (AdV)). ESI-09 caused a significant reduction in the growth of all viruses in treated cells with an average decrease in infectious titers of 3 to 4 log values (Fig. 1, *B* and *C*). Cell viability was measured in cells treated with ESI-09 *via* LDH release assay to confirm that the reductions in viral titers were not a by-product of inhibitor-mediated cellular toxicity, and no significant decrease in viability was observed with ESI-09 up to a concentration of 50 μM (Fig. S1*A*). Brightfield images also showed that infected cells treated with ESI-09 maintain a healthy phenotype with minimal cytopathic effect (Fig. S1*B*). To also confirm that ESI-09 was selectively targeting and inhibiting EPAC in cells, we measured ESI-09–induced changes in the activity of Rap1, a GTPase that is the immediate downstream target of EPAC. Rap1 activity was quantified with a FRET biosensor assay described previously (22) that measures the interaction between GTP-bound (active) Rap1 and its downstream target RalGDS. ESI-09 treatment inhibited activation of Rap1, while 007-AM, an EPAC selective agonist, promoted Rap1 activation (Fig. S1*C*). A dose-response analysis was also performed against VACV and VSV to determine an effective dose-range for ESI-09 treatment (Fig. S1*D*). The inhibitor had an IC_50_ close to 5 μM against both viruses, which, coincidently, is close to the IC_50_ values previously reported for EPAC in cell-free assays (20). It also confirms that the inhibitor is active at a concentration that is both nontoxic and below the range previously reported to result in nonspecific interactions (23).

The stability and potency of ESI-09 antiviral activity was assayed over a 5-day period against the five viruses at three different multiplicities of infection (MOIs) (Figs. 1*D* and S1, *E* and *F*). Measuring the inhibitor activity at different MOIs provides some additional information about the stage of viral life cycle impacted. Low MOIs require multiple replication cycles for detection so it provides information about how the inhibitor modulates virus growth, whereas infections at high MOIs and earlier time points probe more directly for virus entry. At an MOI of 0.1, reporter expression of all viruses, except HSV-1 and AdV, was reduced by more than 90% for the entire duration of the experiment with ESI-09 treatment than their dimethyl sulfoxide (DMSO)-treated counterparts. AdV only surpassed the 10 % mark after four days, while HSV-1 had increased levels of virus replication two days after infection. Similar inhibition for all viruses was observed at an MOI of 1 with only HSV-1 having any appreciable increase in virus replication (Fig. S1*E*). However, this growth was still delayed until two days post infection and did not peak until three days. At a lower MOI of 0.01, all viruses, including HSV-1, were fully inhibited by ESI-09 (Fig. S1*F*). Only AdV exceeded the 10% mark in virus growth at the lower MOI, but the increase was not statistically significant since the level of growth in the DMSO-treated group was also very low.

### ESI-09 is a potent inhibitor of SARS-CoV-2 infection

Prior work has shown that ESI-09 is a potent inhibitor of SARS-CoV-1 and MERS-CoV coronaviruses (21), but it has not been tested against SARS-CoV-2. We investigated whether ESI-09 could inhibit SARS-CoV-2 infection to further demonstrate its therapeutic potential as a broad-spectrum antiviral. In Calu3, Vero-hTMPRSS2, and A549-ACE2 cells, ESI-09 treatment reduced SARS-CoV-2 RNA to almost undetectable levels (Figs. 1*E* and S2). Even when Calu3 cells were infected at an MOI of 1, ESI-09 reduced viral RNA levels by more than 99% (Fig. 1*E*). Infected cells were also stained for expression of the spike protein, which mediates viral attachment and binding (Fig. 1*F*). ESI-09 reduced spike protein expression in Calu3 cells to undetectable levels. Lastly, we examined the efficacy of ESI-09 against three SARS-CoV-2 variants of concern (VOCs, Fig. 1*G*). The alpha, beta, and delta variants all contain mutations within the spike protein, which is the sole target of available vaccines and a major target for SARS-CoV-2 drug testing. Hence, novel mutations within spike are a major concern for drug and vaccine resistance. Regardless, ESI-09 treatment completely inhibited infection of all three SARS-CoV-2 variants (Fig. 1*G*).

### ESI-09 tropism and EPAC isoform selectivity

To characterize the antiviral mechanism of action of ESI-09, while also ensuring that those mechanisms are shared across different families of viruses, we narrowed our analyses to VACV and VSV as representative DNA and RNA viruses, respectively. Since broad-spectrum antiviral protection requires activity against viruses with a variety of tropisms, we investigated the relationship between EPAC expression and ESI-09–induced virus inhibition in a range of immortalized cell lines. There are two mammalian isoforms of EPAC (EPAC1 and EPAC2) that are both equally inhibited by ESI-09. Both isoforms function similarly but vary in tissue and subcellular localization (24, 25). EPAC1 is more ubiquitously expressed throughout a broad range of tissues, while EPAC2 is expressed mainly in the adrenal gland, pancreas, liver, and CNS (24).

EPAC1 and EPAC2 expression was measured in a panel of 20 cell lines by immunoblotting (Fig. S3, *A* and *B*). EPAC2 expression was very low in many of the cell lines, whereas EPAC1 was detectable in almost all cell lines and covered a range of low, medium, and high expression levels (Fig. S3, *A* and *B*). Many of the permissive cell lines that are commonly used to propagate viruses (*i.e.*, U2-OS, Vero, 293T) had very low expression of EPAC1 and EPAC2 (Fig. S3, *A* and *B*). In these cell lines, the level of viral inhibition was also close to 100% (Fig. S3*C*). The cell lines displaying the highest levels of EPAC1 expression were the ovarian cancer lines: SKOV-3, OVCAR-4, AF2028, and AF2068. EPAC1 expression was also high in the pancreatic cell lines, MIA-PaCa-2 and BxPC3, which matches previous findings of high expression in the pancreas (24, 26). In contrast, the cell line with the highest level of EPAC2 expression was SW620, which also had low expression of EPAC1. Regardless of cell line, tissue origin, or level of EPAC1 or EPAC2 expression, ESI-09 inhibited VACV and VSV growth by more than 75% (Fig. S3*C*). We also confirmed that ESI-09 treatment in cell lines with low, medium, or high levels of EPAC1 expression (A549, BxPC3, and OVCAR-4, respectively) reduced infectious titer of VACV and VSV by performing plaque assays (Fig. S3, *D–F*). Similar to the permissive cell lines tested in Figure 1, *B* and *C*, viral titers dropped by at least two log values when cells were treated with ESI-09.

**Figure 2:**
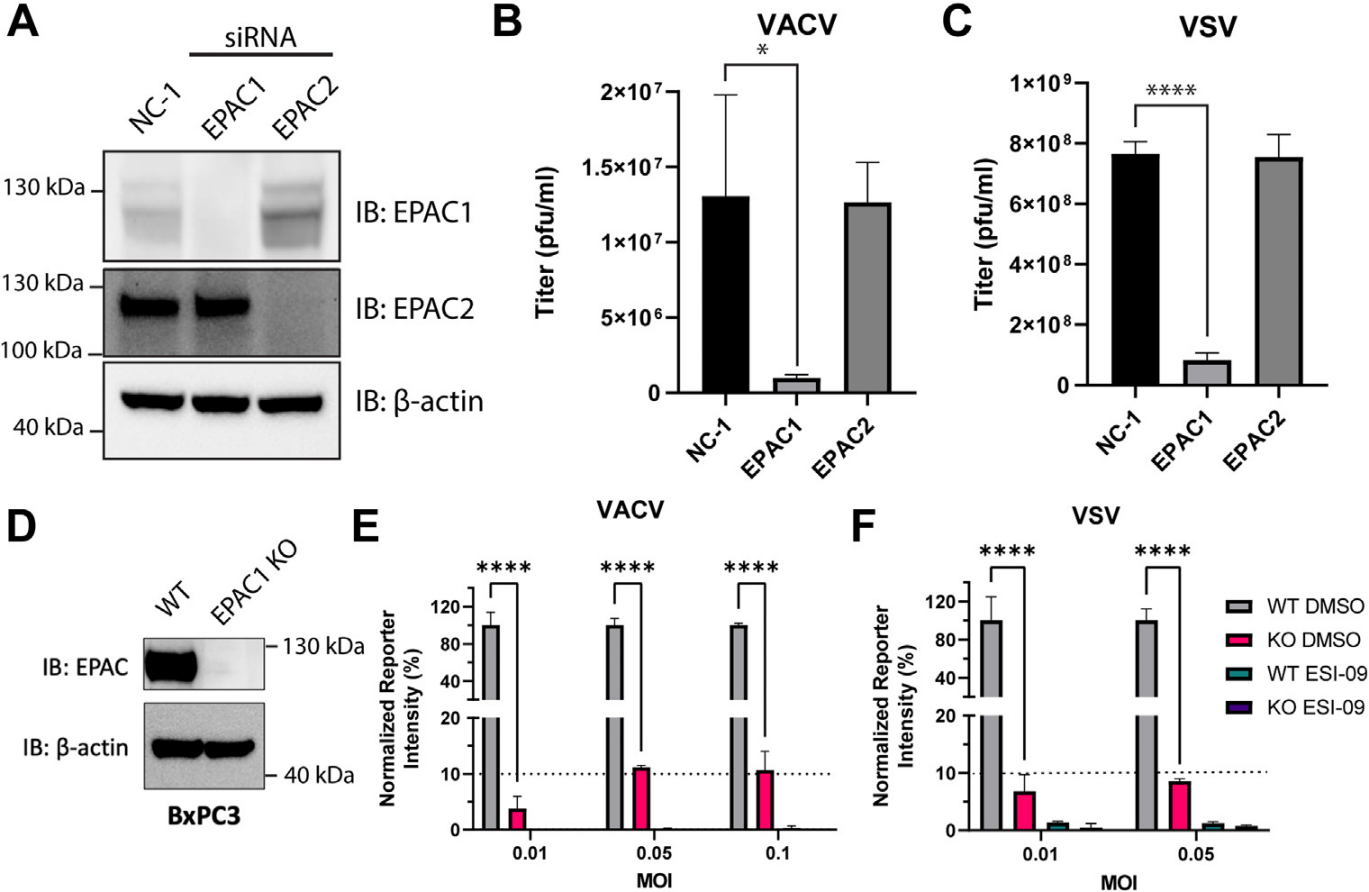
**ESI-09 prevents VACV and VSV infection through inhibition of the EPAC1 isoform.***A*, immunoblot showing EPAC1 and EPAC2 knockdown in 293T cells 48 h after transfection with siRNA. NC-1 reflects scrambled RNA control. *B* and *C*, viral titers for VACV and VSV following infection in cells subjected to siRNA knockdown of EPAC1 or EPAC2. Cells were infected 48 h after transfection of siRNA at an MOI of 0.1 and 0.01 for VACV and VSV, respectively. *D*, immunoblot showing EPAC1 knockout in BxPC3 cells. *E* and *F*, VACV and VSV growth, 24 h post infection, in BxPC3 EPAC1-knockout cells with and without 25 μM ESI-09 treatment. *Dashed lines* reflect the 90% inhibition mark. EPAC, exchange protein activated by cAMP; VACV, Vaccinia; VSV, vesicular stomatitis virus; MOI, multiplicity of infection. Statistical significance determined by Fisher’s LSD test (∗*p* < 0.0361; ∗∗*p* < 0.0021; ∗∗∗*p* < 0.0002; ∗∗∗∗*p* < 0.0001).

Since there were significant differences in the levels of EPAC1 and EPAC2 expression, we tested whether the antiviral activity of ESI-09 was due to selective inhibition of either a single EPAC isoform or the combined inhibition of both isoforms together. We used siRNA to silence EPAC1 or EPAC2 expression prior to infecting with VACV or VSV. Knockdown of the two EPAC isoforms was confirmed by immunoblot (Fig. 2*A*), and virus replication was monitored by both reporter expression and plaque assay. Knockdown of EPAC1 resulted in a 10-fold reduction in viral titer for both VACV and VSV, whereas EPAC2 knockdown had no significant effect on virus replication (Figs. 2, *B* and *C* and S4).

To further probe the EPAC1 selectivity of ESI-09, we created EPAC1 KOs in cells with CRISPR/Cas9 (Fig. 2*D*). We tried to generate KOs in BxPC3 and OVCAR-4 cells, since they are cells of different tissues’ origin that both express high levels of EPAC1. However, we were unable to rescue the OVCAR-4 EPAC1 KO line. In EPAC1-KO BxPC3 cells, we measured VACV and VSV replication in combination with ESI-09 treatment (Fig. 2, *E* and *F*). EPAC1 knockout in BxPC3 cells caused a 90 to 96% reduction in replication of VACV and VSV. In contrast, ESI-09 treatment of WT BxPC3 cells reduced virus growth by roughly 98 to 99%. ESI-09 treatment with the knockout cells led to a similar level of inhibition. Despite the evidence that EPAC1 is the dominant isoform that mediates ESI-09’s antiviral activity, there may be a smaller dependence on EPAC2 inhibition as well. Since EPAC2 was proposed as the dominant isoform required for inhibition of RSV (27), the EPAC1 *versus* EPAC2 selectivity may ultimately depend on their relative level of expression in the target cells.

### ESI-09 has a multimodal mechanism of action that impacts virus entry and spread

**Figure 3:**
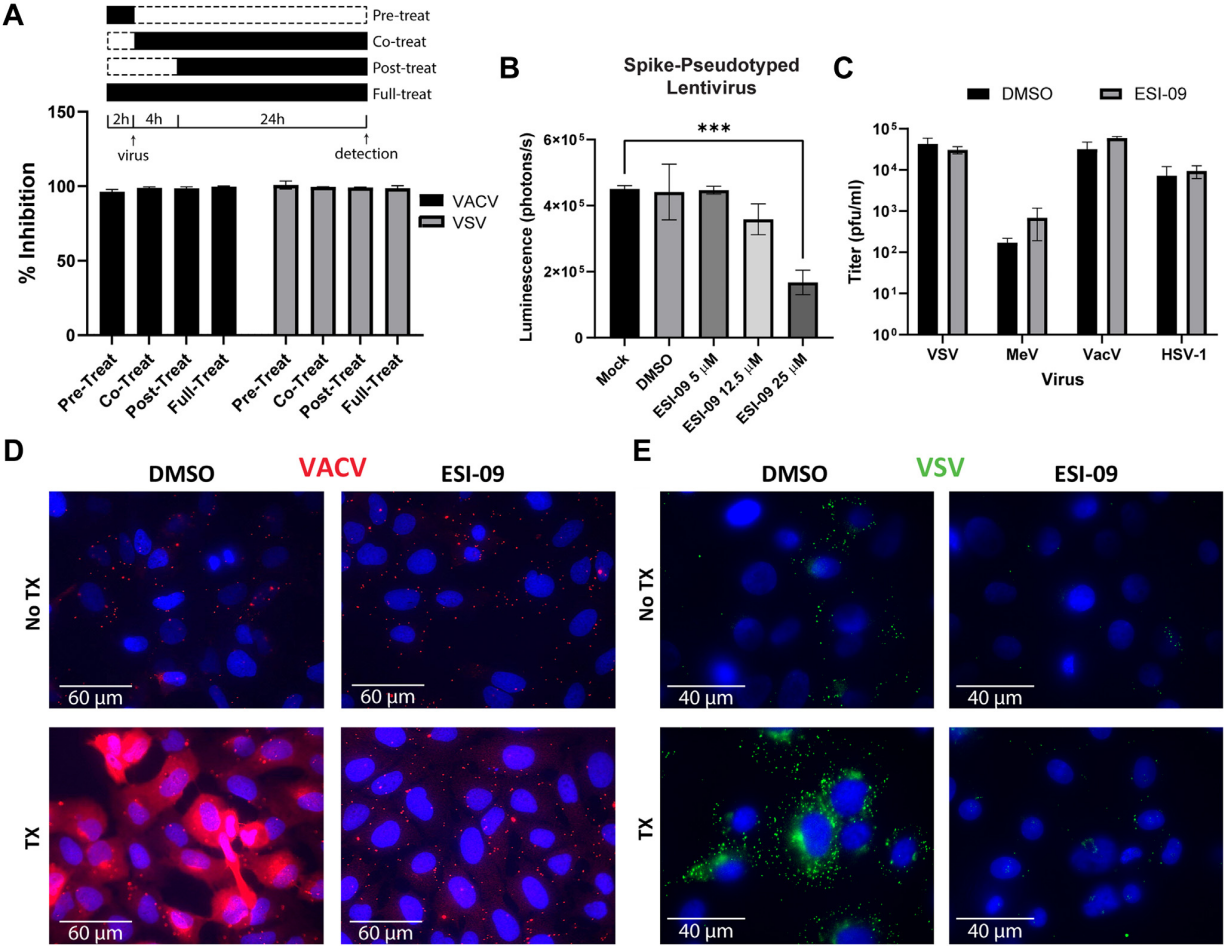
**ESI-09 inhibits VACV and VSV internalization.***A*, analysis of ESI-09 virus inhibition with pretreatment and posttreatment. The schematic above the plot describes the treatment regimen of ESI-09 (*solid black bars*) with respect to virus infection. The concentration of ESI-09 used for each treatment regimen was 25 μM. Percent inhibition was measured by firefly luciferase activity for VACV and GFP for VSV, normalized relative to the DMSO control and background signal. *B*, ESI-09 dose-dependent inhibition of SARS-CoV-2 spike-pseudotyped lentivirus into Vero-E6 (ACE2/TMPRSS2) cells. *C*, measurement of virus attachment/entry to Vero cells after coincubation with virus particles at an MOI of 20 for 1 (VSV, MeV) to 2 (VACV, HSV-1) hours. Cells were pretreated with 25 μM ESI-09. Following coincubation, cells were repeatedly washed with PBS and then isolated to titer virus particles that were attached or internalized. *D* and *E*, immunofluorescence of VACV (*D*) and VSV (*E*) proteins after infection with an MOI of 10 for 2 h. Cells were then either stained directly with VACV or VSV antibodies (No TX) to look at viral attachment or permeabilized prior to staining with triton-x 100 (TX) to observe virus entry into cells. HSV, herpes simplex virus-1; MeV, measles virus; MOI, multiplicity of infection; VACV, Vaccinia; VSV, vesicular stomatitis virus. Statistical significance determined by Fisher’s LSD test (∗*p* < 0.0361; ∗∗*p* < 0.0021; ∗∗∗*p* < 0.0002; ∗∗∗∗*p* < 0.0001).

To determine which stage of the virus life cycle was impacted by ESI-09, we applied different treatment regimens in which the inhibitor was added to cells prior to, concurrently, or following virus infection. Interestingly, under all treatment regimes, VACV and VSV inhibition was close to 100% (Fig. 3*A*). There were some subtle, but not statistically significant, differences between the treatment strategies with posttreatment and pretreatment (ESI-09 removed during infection) groups having slightly less inhibition than treatments where ESI-09 was present at all points of virus infection, but overall, the results suggest that ESI-09 inhibits multiple stages of the VACV’s and VSV’s life cycles.

The antiviral activity observed with pretreatment of cells suggests that one mechanism of ESI-09 action is the blocking of virus attachment or entry, which has also been proposed previously for Ebola virus (28). We therefore examined if ESI-09 could prevent nonreplicating viruses from infecting cells. Vero-E6 cells expressing ACE2 and TMPRSS2 were treated with ESI-09 and then incubated with a replication-incompetent lentivirus pseudotyped with SARS-CoV-2 spike protein and encoding luciferase. After 24 h, luciferase activity was measured (Fig. 3*B*). Cells treated with ESI-09 saw more than a 50% reduction in luciferase signal. We performed an additional experiment with a nonreplicating AdV with an early region 1 (E1) gene deletion and similarly found that ESI-09 reduced levels of AdV-expressed mCherry by ∼60 to 90% (Fig. S5). To determine if ESI-09 prevented virus attachment/entry to cells, we incubated different viruses with target cells for 1 to 2 h to allow binding, washed the cells to remove unbound virus, and then titered the amount of virus either adhered to or internalized within cells (Fig. 3*C*). The treatment of cells with ESI-09 led to no significant reduction in viral attachment in contrast to cells treated with DMSO alone.

Inhibition of virus entry by ESI-09 treatment was further characterized through immunofluorescence experiments using a treatment strategy similar to the binding experiment described above. VACV or VSV were incubated with cells following a pretreatment period with ESI-09 or vehicle control, and after 1 to 2 h, cells were fixed and stained with anti-VACV or anti-VSV antibodies. Staining without permeabilization resulted in detection of small, isolated spots scattered around the cells (Fig. 3, *D* and *E*, top panels). When cells were permeabilized with triton-x 100 during staining, to enable antibody binding within cells, fluorescence signal was spread throughout the cells in the vehicle control group, but not in the ESI-09 group (Fig. 3, *D* and *E*, bottom panels). For both viruses, the permeabilized ESI-09 samples appeared similar to the nonpermeabilized samples with only scattered isolated spots. These results are consistent with ESI-09 inhibiting entry but not limiting attachment of VACV or VSV.

**Figure 4:**
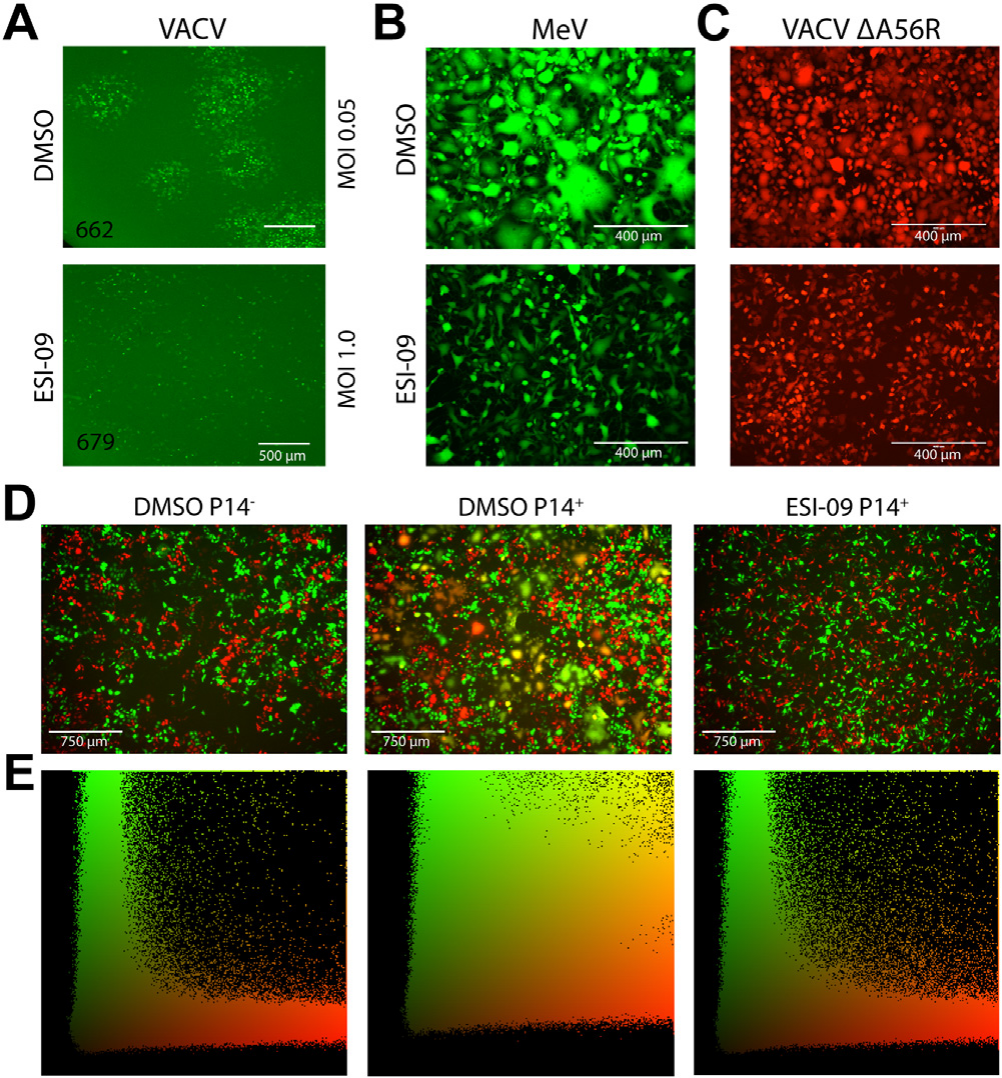
**ESI-09 prevents both syncytia formation induced by VACV, MeV, and the P14 FAST protein.***A*, comparison of VACV plaque formation with and without ESI-09 treatment in U2OS 24 h post infection. The numbers in the bottom left indicate the number of GFP-positive cells in each sample. ESI-09–treated cells were infected at a higher MOI to establish an infection with inhibitor present that gave similar numbers to the DMSO control. *B* and *C*, ESI-09 induced inhibition of syncytia caused by MeV in Vero cells (*B*) or VACV-ΔA56R in U2OS cells (*C*). *D*, images of cell fusion mediated by the transfection of P14 glycoprotein pcDNA in ESI-09 *versus* DMSO-treated HEK293T cells. *E*, colocalization scatterplots for GFP and mCherry from the images in panel D. MeV, measles virus; MOI, multiplicity of infection; VACV, Vaccinia.

The postinfection treatment regimen (Fig. 3*A*) indicated that ESI-09 could also inhibit VACV and VSV growth even when given several hours after exposure, at a time when the viruses would have already entered cells. Hence, there is likely a secondary mechanism of action for ESI-09’s antiviral activity that targets a later part of their life cycles. When ESI-09–treated cells are infected with a sufficiently high MOI of virus or if left for longer periods of time, it is possible to establish virus infection. However, under these conditions, there are significant changes in the morphology of infected cells treated with ESI-09. For instance, a conventional VACV infection creates circular plaques in monolayers due to cell-to-cell viral transmission (Fig. 4*A*, top). With ESI-09 treatment, traditional plaque formation is abrogated, resulting in a disperse infection (Fig. 4*A*, bottom). This suggests that ESI-09 treatment is capable of disrupting the cell-to-cell transmission of VACV.

Syncytia formation (cell fusion) is a common mechanism utilized by viruses to improve infection through cell-to-cell spread of virus particles or genomes (29, 30). In previous work, Choi *et al*. found that EPAC inhibition prevented syncytia formation from RSV infection (27). Here, we explored whether ESI-09 could disrupt syncytia formation by two fusogenic viruses: MeV, which induces syncytia formation *via* its F and H glycoproteins, and VACV-ΔA56R, whose knockout prevents inhibition of the VACV fusion complex that mediates virus entry (31, 32, 33). In DMSO-treated cells, both MeV and VACV-ΔA56R cause extensive fusion between cells, whereas in ESI-09–treated cells, syncytia is either substantially reduced or abrogated altogether (Fig. 4, *B* and *C*). It was unclear whether ESI-09 treatment blocked syncytia directly or as a consequence of another antiviral pathway that inhibited virus growth. To address this, we examined whether ESI-09 could inhibit syncytia formation, in the absence of virus, from the transfection of the P14 fusion-associated small transmembrane peptide from orthoreovirus (34, 35). Cell fusion was monitored by mixing cells expressing either mCherry or eGFP and measuring colocalization of their fluorescence. In untreated cells that were not transfected with P14, the mCherry and eGFP signals were clearly separated and the resulting colocalization scatterplot showed that there was little to no overlap of their fluorescence (Fig. 4, *D* and *E*). In contrast, untreated cells transfected with P14 exhibited extensive fusion as seen by the formation of large multinucleated cells with overlap of green and red fluorescence. The resulting colocalization scatterplot revealed a large number of objects coexpressing red and green fluorescent proteins. Cells treated with ESI-09 and transfected with P14 displayed a very similar phenotype to the untransfected control cells with almost no colocalization of red and green signals. ESI-09 is therefore capable of directly blocking virus-induced syncytia.

### ESI-09 inhibits viral entry and spread *via* remodeling of actin filaments

EPAC is a signaling protein that regulates a wide variety of physiological processes that could be responsible for inhibiting virus infectivity. To identify the downstream pathways involved in virus entry and spread that are impacted by ESI-09 treatment, we examined cellular machinery involved with both viral entry/endocytosis and cell fusion. The viruses tested in this study employ a variety of entry strategies ranging from fusion at the cell membrane mediated through viral proteins (MeV) to receptor-mediated endocytosis *via* clathrin or caveolin pits (VSV, AdV) to micropinocytosis (VACV, HSV) (36, 37, 38). One consistency between these strategies is their reliance on actin filaments (39). Similarly, viral transfer through direct cell-to-cell mechanisms is also controlled *via* actin (29, 30). We examined if the infection of cells with our panel of viruses (Fig. 1*B*) induced changes in actin filaments by staining with a fluorescent phalloidin dye and found that all viruses induced some change in actin structures (Fig. S6). VSV caused increases in cortical actin, while decreasing cellular actin. MeV led to dramatic decreases in actin levels in cells that had undergone syncytia. VACV created clusters of globular actin and long filamentous nanotubes between cells, and HSV-1 caused cell rounding with an overall increase in cortical actin. Despite some variability from virus to virus, these results show that actin is an important component that is modulated during virus replication and thus a promising target that might influence virus infection.

**Figure 5:**
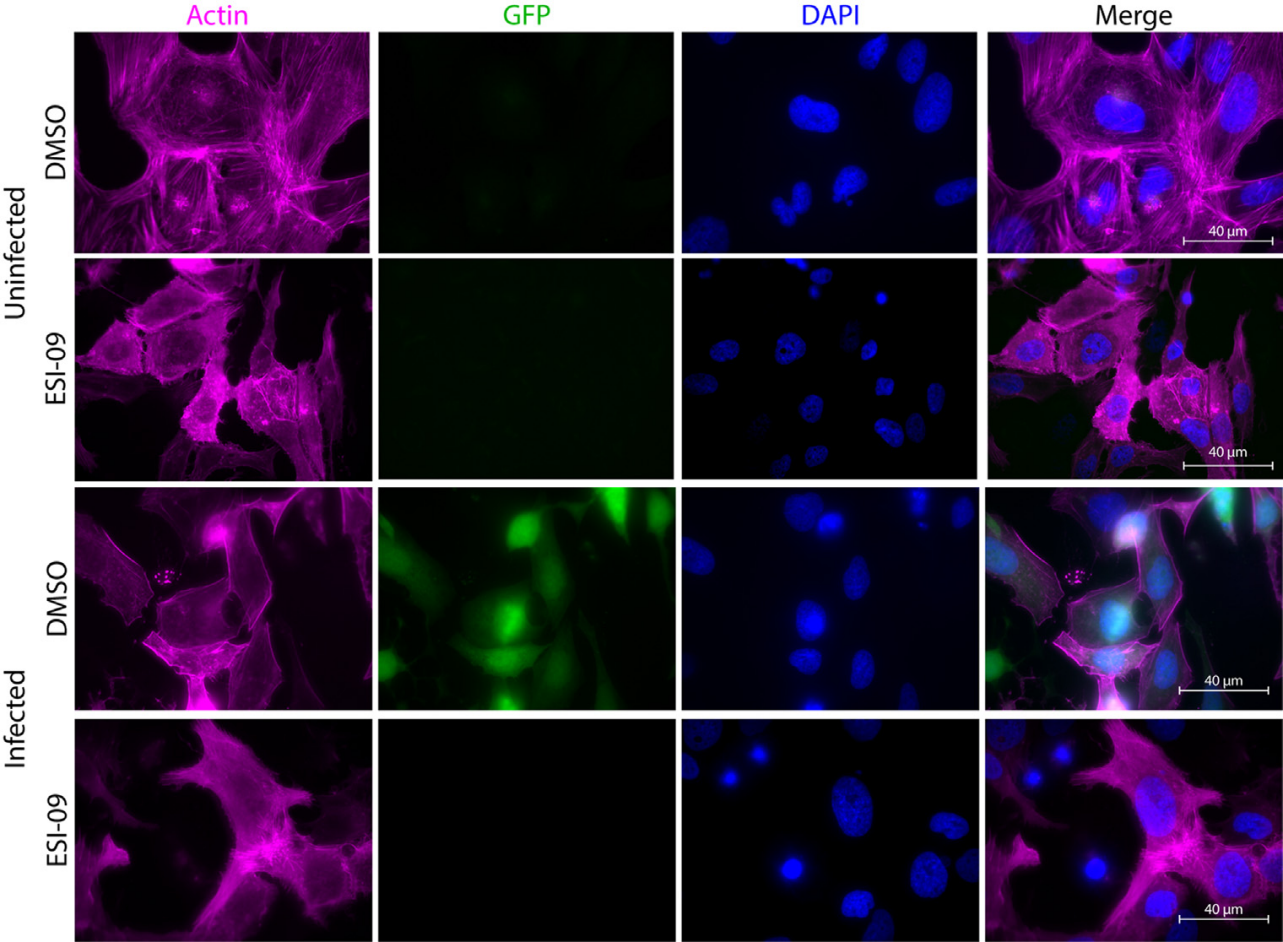
**ESI-09 remodeling of actin filaments in VSV-infected cells.***A*, phalloidin staining of actin filaments (*magenta*) in Vero cells treated with ESI-09 and infected with VSV (*green*). Samples were stained 24 h after infection/treatment. VSV, vesicular stomatitis virus.

EPAC has a well-established role in actin remodeling that occurs through downstream regulation of Rho family GTPases (14, 16, 40). To determine if ESI-09–induced EPAC inhibition impacted the actin cytoskeleton in infected cells, we stained uninfected and VSV-infected cells with phalloidin (Fig. 5). In uninfected cells, ESI-09 led to remodeling of the actin cytoskeletal network, with a noted decrease actin around the nucleus of cells. As noted above, infection with VSV in untreated cells increases cortical actin around the edges of the cells and decreases cytosolic actin. In contrast, ESI-09–treated cells reduce cortical actin at the cell edges and induce formation of actin stress fibers positioned more consistently throughout the cell. However, there is also less actin around the nucleus as similarly observed for ESI-09 treatment in uninfected cells.

To confirm that changes in the actin cytoskeleton were due to EPAC1 inhibition, we stained the BxPC3 EPAC1 KO cells with Phalloidin (Fig. S7). Both WT BxPC3 cells treated with ESI-09 as well as untreated EPAC KO cells exhibited a similar change in actin morphology relative to untreated WT BxPC3, noted by a decrease in actin staining around the nucleus. Although the change is slightly different than that observed in Vero cells (Fig. 5), the results confirm that ESI-09 acts on EPAC to induce cytoskeleton remodeling in cells.

### EPAC-mediated actin remodeling facilitated *via* Rho GTPases and Arp2/3 complex

**Figure 6:**
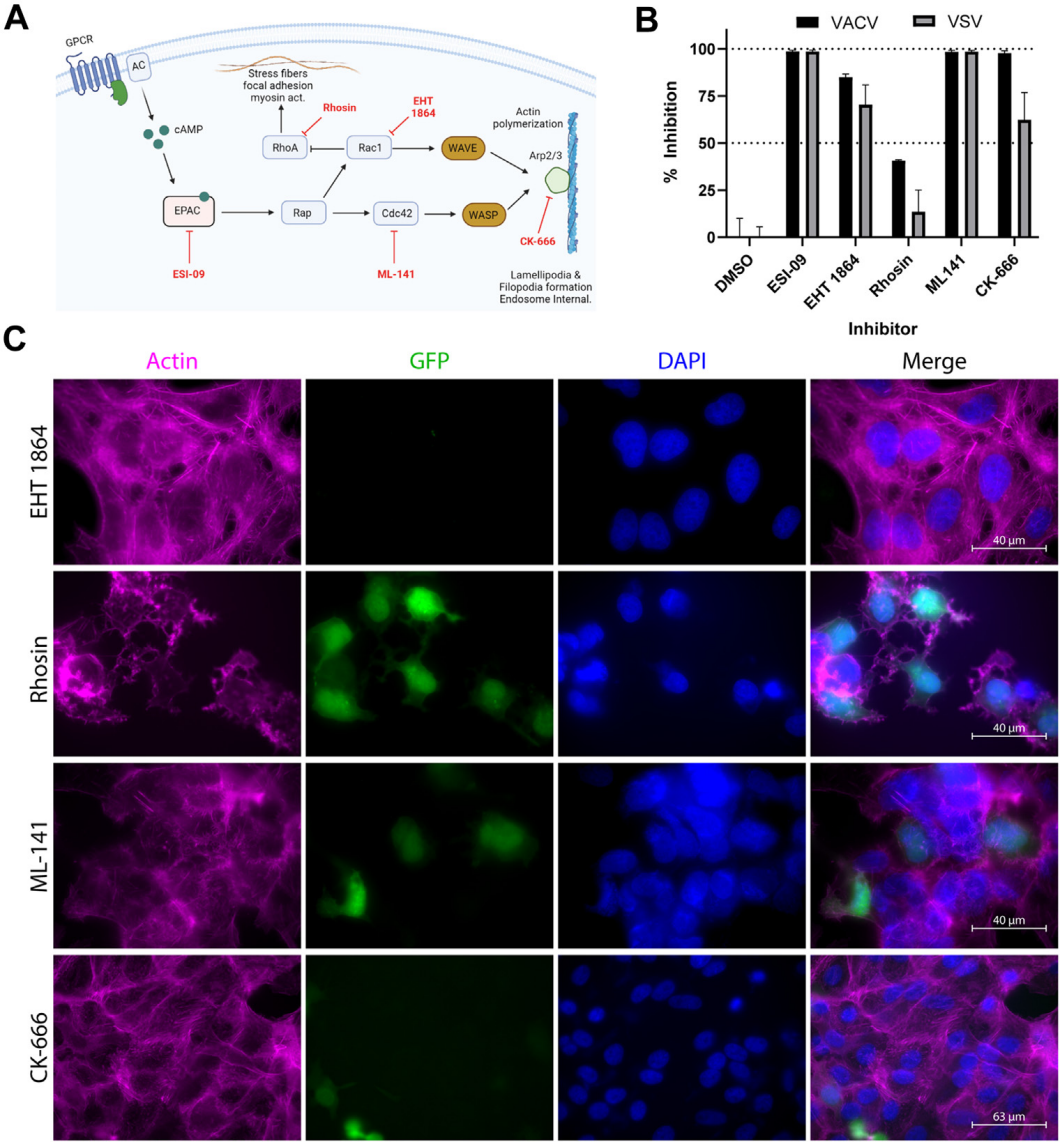
**ESI-09 antiviral activity mediated *via* Rho GTPases and Arp2/3 complex.***A*, schematic of EPAC signaling pathways in the regulation of actin dynamics. The *red bars* denote inhibitors of their respective targets. *B*, inhibition of VACV and VSV in cells treated with GTPase inhibitors. *C*, phalloidin staining of actin filaments in Vero cells treated with GTPase inhibitors. EPAC, exchange protein activated by cAMP; VACV, Vaccinia; VSV, vesicular stomatitis virus.

EPAC is known to regulate actin remodeling *via* Rap1 (16), which consequently acts on Rac1 (40, 41) and Cdc42 (42) GTPases (Fig. 6*A*). Rac1 and Cdc42 GTPases activate WAVE and WASP, respectively, which promote formation of the Arp2/3 complex that serves as a nucleation site for actin polymerization (43). Actin polymerization mediated by the Arp2/3 complex is known to play a role in lamellipodia and filopodia formation as well as endosome internalization, which are all important for viral attachment and entry (41, 44). Inhibition of Rac1 results in activation of RhoA, a GTPase that promotes stress fiber formation (45), which explains the increased levels of stress fiber formation in ESI-09–treated cells. To determine if the Arp2/3 pathway was responsible for ESI-09–mediated viral inhibition, we tested whether inhibitors of Rac1 (EHT-1864), Cdc42 (ML-141), RhoA (Rhosin HCl), or Arp2/3 (CK-666) could similarly inhibit VACV and VSV replication (Fig. 6*B*) (46, 47, 48, 49). ML-141 fully inhibited VACV and VSV to a level similar to ESI-09. EHT-1864 and CK-666 were also effective inhibitors of virus replication although to a lesser degree than ESI-09 or ML-141. Rhosin was the weakest inhibitor of all the compounds tested with only a 13% reduction in virus growth for VSV and a 40% reduction for VACV. ML-141, EHT-1864, and CK-666 all exhibited dose-dependent inhibition of VACV and VSV with IC50s in the 10 to 20 μM range, whereas Rhosin had either very weak or negligible inhibition over the same range (Fig. S8, *A* and *B*). In addition, none of these inhibitors, at any of the concentrations tested, had any significant cytotoxicity towards the cells (Fig. S8*C*).

Actin staining was performed on cells treated with the GTPase inhibitors to determine whether they remodeled actin filaments in a similar manner to ESI-09 (Fig. 6*C*). Both EHT-1864 and ML-141 promoted stress fiber formation similar to ESI-09, whereas Rhosin treatment led to very different phenotype marked by low levels of stress fibers. We also repeated the colocalization experiment with the P14 fusogenic protein in the presence of the GTPase inhibitors to determine whether they could similarly inhibit syncytia formation (Fig. S9). All inhibitors reduced syncytia formation by varying degrees. CK-666 caused the largest reduction of syncytia that was second only to ESI-09, while Rhosin caused only a minor reduction in cell fusion and overall had a very similar phenotype to the DMSO-treated sample. Altogether, the findings support a mechanism for ESI-09 *via* inhibition of downstream GTPases involved in actin remodeling.

### ESI-09 inhibits VACV replication in murine lungs *ex vivo* and *in vivo*

**Figure 7:**
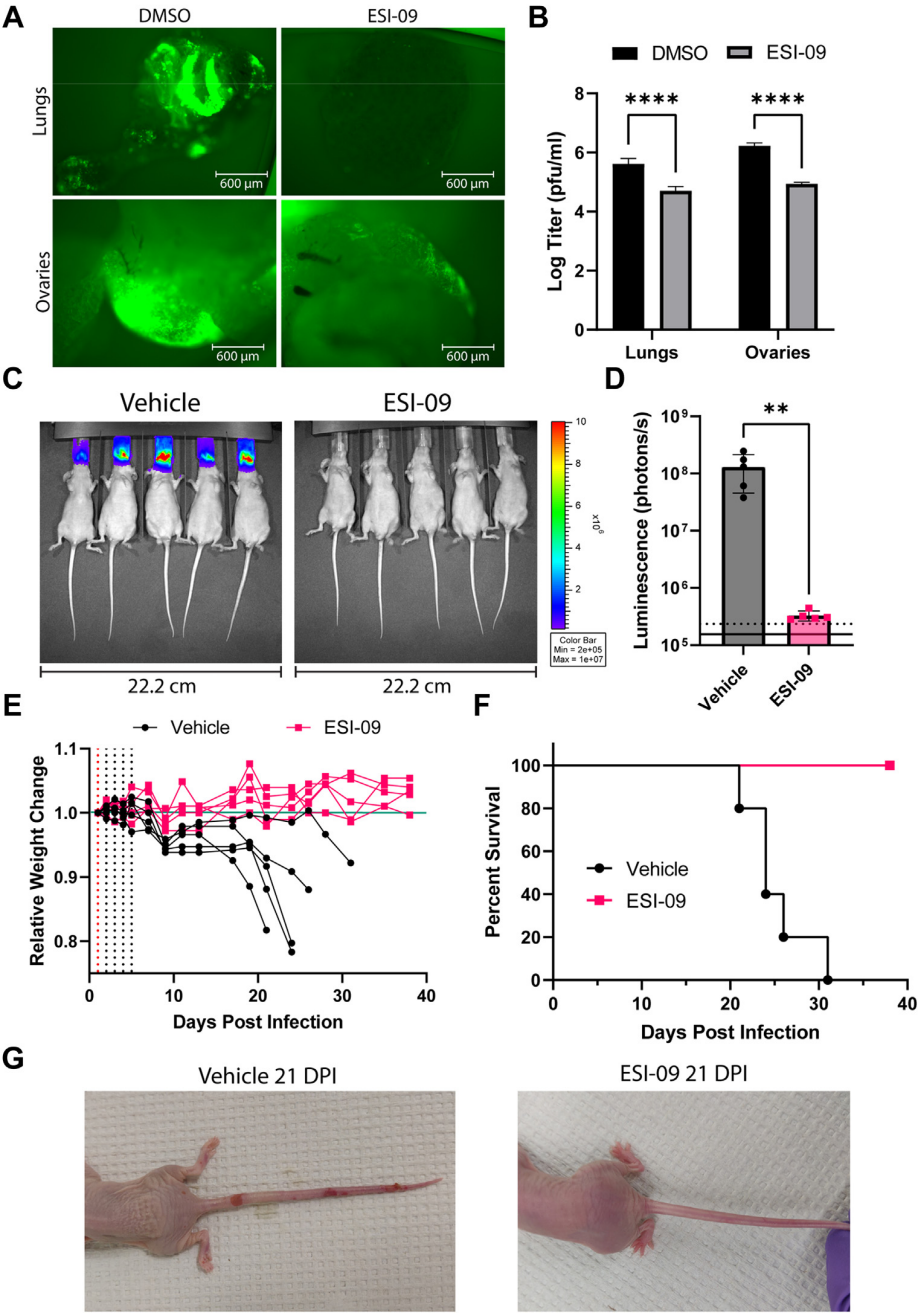
**ESI-09 prevents VACV infection in *ex vivo* murine tissues and immune-deficient mice.***A*, lungs and ovaries were extracted from Balb/c mice, processed into 2 mm cores, and infected with VACV with and without ESI-09 treatment. Fluorescent images were acquired three days after infection to monitor virus replication *via* virus-expressed GFP. *B*, Plaque assay results from infected tissues from panel *A*. *C*, IVIS imaging showing VACV-induced luciferase activity four days after infection in nude mice after ESI-09 treatment. Mice received 36 μg/kg/d ESI-09 or vehicle for five days starting on the same day of infection. *D*, luminescence intensities from panel *C*. Background signal is depicted by the *solid black line*, while the background + 5 standard deviations is depicted by the *dashed line*. *E*, relative weight loss in mice infected with VACV Tiantan strain intranasally while also receiving 36 μg/kg/d ESI-09 or vehicle. *Dashed lines* indicate treatment dates for the inhibitor, while the *red line* indicates the date when virus was administered. *F*, survival plot for mice from panel *E*. *G*, representative images of tail pox lesions in mice 21 days after infection. VACV, Vaccinia.

Cell-to-cell viral transmission is important *in vivo* for efficient spreading and evasion of host defenses. As an initial step to determine if ESI-09 could inhibit virus replication in complex three dimensional tissues, we removed lungs and ovaries from mice and infected them with VACV in the presence of the inhibitor. The lungs were processed into 2 mm cores, while the ovaries were used directly from the mice. In both lung cores and ovaries treated with ESI-09, there was a drastic reduction in VACV replication and an overall reduction in viral spread (Fig. 7, *A* and *B*). We also measured ESI-09 antiviral activity in human tissue samples taken from cancer patients and similarly found a reduction in viral growth in ESI-09–treated tissues (Fig. S10, *A* and *B*). In addition, ESI-09–treated samples had smaller reductions in viability following infection than untreated VACV-infected samples (Fig. S10*C*).

With promising results against murine and human tissue samples, we next investigated whether ESI-09 treatment could be applied *in vivo* to protect mice from VACV infection. We developed a VACV challenge model in CD1 nude mice using intranasal (IN) administration of both the virus and inhibitor. To determine an effective dose, mice were injected IN with three different concentrations of ESI-09 for five consecutive days and monitored for signs of inhibitor-induced toxicity. At all three doses, there were no significant changes in weight relative to the vehicle control (Fig. S11). However, at the higher doses, there were some issues with solubility, so we chose the lowest dose to test inhibitory efficacy *in vivo*. Mice were treated with 36 μg/kg ESI-09 on the same day they were infected with VACV and on each of the four following days. On the final day of treatment (four days after infection), virus replication was measured *in vivo* by IVIS (Fig. 7, *C* and *D*). There were high levels of luciferase activity in all five of the control mice indicating high levels of virus replication, whereas no significant luminescence was detected in all mice of the ESI-09–treated group. Weight change in infected mice was tracked for 40 days to monitor disease progression (Fig. 7*E*). The vehicle-treated group experienced a drop (∼6%) in body weight eight days post infection, which leveled off over the following few days before rapidly decreasing again around day 20. Around this time, mice in the vehicle control group began developing dry scaly skin and pox lesions in their tails and lower backs (Fig. 7*G*), as well as general signs of severe infection such as lethargy and temperature loss. Due to these symptoms and their rapid weight loss, all mice within the vehicle group required euthanization. In contrast, the ESI-09–treated group had only a slight drop (∼2–3%) in body weight after eight days, which they quickly recovered from (Fig. 7*E*). For the remainder of the experiment, they maintained a body weight at or above their original mass. They also never exhibited any symptoms of severe VACV infection, such as pox lesions, scaly skin, or pallor, suggesting that ESI-09 either completely prevented infection or drastically reduced viral burden so that the mice could make a complete recovery following their initial exposure. Altogether, these findings suggest that ESI-09 could be a promising therapeutic against pathological poxvirus infection.

## Discussion

The SARS-CoV-2 and mpox outbreaks have highlighted the need to expand our arsenal of antiviral countermeasures. We investigated EPAC antagonism as a strategy for broad-spectrum antiviral development in this study and found that the EPAC selective inhibitor, ESI-09, elicited antiviral activity against a variety of viruses, including the SARS-CoV-2 coronavirus and VACV, an orthopox virus from the same genus as the mpox virus. We demonstrate that ESI-09 can inhibit both RNA and DNA viruses, as well as enveloped and nonenveloped viruses. It is also active in a variety of cell types, making it effective against a wide range of virus tropisms and it provides long-term activity in cell culture with minimal toxicity. We show that ESI-09 regulates internalization and cell-to-cell transmission of VACV and VSV *via* extensive remodeling of actin filaments. As a result, the inhibitor blocks VACV and VSV at various stages of their life cycles.

EPAC has a well-established role in the regulation of actin dynamics, which it mediates through a series of GTPases (summarized in Fig. 6*A*). EPAC activation modulates barrier function in endothelial cells by increasing cortical actin *via* an Rac1 GTPase-dependent pathway (16, 40). In contrast, knockdown of EPAC with siRNA lowers cortical actin and increases stress fiber production in a manner similar to what we observed with ESI-09 treatment (16). We found that inhibiting Rac1, Cdc42, or the downstream Arp2/3 complex suppresses VACV and VSV replication in a similar manner to ESI-09. Inhibition of these targets also results in a decrease in cortical actin and an overall increase in actin stress fibers. The actin network changes are comparable to those that were previously seen with RhoA activation (45, 50). In contrast, RhoA inhibition, which decreases actin stress fibers, exhibits only a minor inhibitory effect on virus replication.

The ESI-09–induced remodeling of actin filaments influences a variety of internalization pathways that are utilized for virus entry. VSV and AdV are internalized *via* clathrin-mediated endocytosis, which uses actin to form invaginations that envelop the virus into endosomes. Cdc42 is integral to this process as it serves to activate the Arp2/3 complex *via* N-WASP and promote actin polymerization (51). VACV and HSV are internalized through macropinocytosis, which is facilitated by plasma membrane protrusions generated from actin filaments–a process that is mediated in part by Rac1 activation but also requires RhoA deactivation (52). Even viruses that directly mediate fusion for entry rely on actin cytoskeleton components. HIV’s envelope protein, which facilitates viral attachment and entry, activates Rac1 to stimulate remodeling of actin networks prior to membrane fusion (53). Ultimately, the ubiquitous role of actin in host and virus internalization processes demonstrates why the inhibition of EPAC and the downstream GTPases that regulate actin dynamics is such an effective strategy for preventing viral infection.

The secondary mechanism of ESI-09 also prevents the virus from spreading beyond the primary site of infection. We show that ESI-09 disrupts traditional plaque formation and suppresses both virus- and P14-mediated cell fusion. Inhibitors of Rac1, Cdc42, and Arp2/3, but not RhoA, could similarly suppress P14-induced syncytia. It was also previously known that the p14 protein hijacked the actin cytoskeleton *via* an N-WASP/Arp2/3–dependent pathway (34). Even without causing syncytia, viruses exploit a variety of actin-controlled pathways to spread between cells. Filopodia that are generated from Cdc42 signaling (52, 54) can create intercellular bridges that viruses can migrate along, either internally or externally, to infect new target cells (30, 55). Cadherins that depend on EPAC, Rac1, and Arp2/3 signaling to assemble and mature (56, 57, 58) also promote cell-to-cell transmission by mediating adhesion between cells (30, 59, 60). Heterologous expression of E-cadherin in HSV has been shown to promote syncytia and increase virus spread in an otherwise nonfusogenic virus (61).

Although our primary focus was on internalization and transmission pathways, ESI-09 could potentially inhibit virus replication through a variety of additional mechanisms, especially since actin can impact practically every stage of the viral life cycle (17). For instance, actin is involved in the egress of many viruses. For VACV, actin helps transport certain isoforms of the virus to the plasma membrane where it recruits additional actin polymerization machinery to launch it from the cell surface (62, 63). Similarly, chemical stabilization or disruption of actin filaments prevents budding and release of MeV from the plasma membrane (64). Other mechanisms such as immune regulation or inhibition of viral protein translation have also been proposed but were not considered in this study (21, 27).

Actin is an intriguing target for antiviral drug development given its roles in virus entry, egress, and spread (17). There are many examples of compounds that modulate actin dynamics that elicit antiviral activity (34, 53, 65, 66, 67). Actin drugs have also been highly sought after for cancer therapy (68, 69). There are many aspects of cellular transformation and changes in actin dynamics that are similar between cancer and virus-infected cells (17). Actin-regulated processes, such as filopodia production or cellular adhesion, that are exploited by viruses to enter and spread within cells are also often important for processes like cancer cell migration and invasion (17, 68). In several prior studies, ESI-09 has been presented as a promising drug candidate for the treatment of pancreatic cancer (19, 20). Similarly, EHT-1864, ML-141, and CK-666 were all previously evaluated as drugs against cancer because of their ability to reduce cell migration (46, 47, 48).

The current challenge with actin-targeted therapeutics is their toxicity. Actin is ubiquitously expressed throughout the body and important for many processes in normal cells. This is why there are currently no approved drugs that target actin directly. However, indirect targeted modulation of actin *via* upstream signaling proteins may provide a viable alternative. As we have seen in this study, indirect targeting of the actin cytoskeleton with ESI-09 and the GTPase inhibitors was well tolerated with minimal toxicity both *in vitro* and *in vivo*. This approach may serve as a less toxic strategy for regulating actin dynamics since signaling proteins are regularly shuttled between inactive and active states depending on their environment, whereas actin stabilizers and destabilizers act more abrasively in modifying the actin cytoskeleton.

In this study, we also demonstrate the therapeutic potential of EPAC inhibition for treatment of SARS-CoV-2 infections. The emergence of novel SARS-CoV-2 variants has exacerbated the pandemic and created uncertainty about the efficacy of vaccines, which currently use the spike protein with the original Wuhan sequence as immunogen. At this time, five VOCs and two variants of interest as defined by the WHO (https://www.who.int/en/activities/tracking-SARS-CoV-2-variants/), all contain extensive mutations within the spike protein. Especially concerning is the Omicron variant with 35 point mutations in spike. Spike mutations increase the risk of vaccine resistance (70, 71, 72), so establishing antivirals that prevent SARS-CoV-2 infection *via* host-targeted mechanisms provides promising treatment options that are less susceptible to the evolution of viral resistance. Here, we show that ESI-09 provides protection against the alpha, beta, and delta variants of SARS-CoV-2. It therefore serves as a promising therapy for COVID-19 that is less susceptible to SARS-CoV-2 mutations that may result in vaccine resistance. In addition, syncytia formation by SARS-CoV-2 in severe infections is associated with extensive lung damage that can lead to chronic respiratory disease (73, 74, 75). It can also provide a means for the virus to evade antibodies produced from vaccination (75). As we have demonstrated in this study, ESI-09 is a potent inhibitor of syncytia formation. It could therefore provide a promising treatment option to reduce the severity and long-term complications of SARS-CoV-2 infection.

In addition to the COVID-19 pandemic caused by SARS-CoV-2, there have been numerous outbreaks over the past few decades from other viruses, such as influenza, Ebola, and poxviruses. mpox is currently spreading rapidly around the world and has been declared a global health emergency by the WHO. While there are effective vaccines and antivirals available against mpox and other orthopox viruses, the recent outbreak has reemphasized how quickly viral diseases can spread throughout the world and why it is important to have an arsenal of antivirals and vaccines ready to meet emerging threats.

Virus-specific antiviral strategies, which comprise the majority of available antiviral drugs, have good safety and efficacy due to their selectivity but also lack versatility and are more susceptible to resistance arising from mutations (3, 76). In contrast, broad-spectrum antivirals, especially those that are host-targeted, lack selectivity, but are effective against a variety of viruses, have a low likelihood of resistance development, and are easy to get secondary approval against new targets once initially validated. The latter category, which ESI-09 falls under, provides a practical and promising strategy to develop “off-the-shelf” therapeutics to combat future pandemics.

## Experimental procedures

### Viruses and cell lines

VACV Copenhagen strain was obtained from American Type Culture Collection (ATCC). Firefly luciferase and eGFP were inserted into VACV at the B14R locus under the control of an early/late promoter as described previously (33). VACV was cultured and titered on U2OS cells using previously described methods (33). VSVΔ51-expressing eGFP was used in this study. It was cultured and titered in Vero cells as previously described (77). HSV1-eGFP (KOS strain) was a gift from Dr Karen Mossman (McMaster University). It was also cultured and titered in Vero cells using previously described methods (78). The MeV-expressing GFP (Schwarz strain) was a gift from Dr Guy Ungerechts (Ottawa Hospital Research Institute).

AdRP3089 has been described previously (79) and is a replication competent AdV type 5 that contains a monomeric red fluorescent protein (RFP) coding sequence with an upstream splice acceptor site replacing the viral early-region 3, which places RFP expression under the control of the viral major late promoter. Thus, AdRP3089 only expresses appreciable levels of RFP late during the virus life cycle and only if the virus undergoes active replication. AdRP3165 is an E1/early-region 3–deleted AdV type 5–based vector that encodes mCherry fluorescent protein under regulation by the human cytomegalovirus immediate early enhancer/promoter and bovine growth hormone polyadenylation sequence and was constructed using standard techniques (80). Both AdRP3089 and AdRP3165 were propagated on 293 cells, purified by cesium chloride buoyant density centrifugation, and titered using standard techniques (80).

The different SARS-CoV-2 isolates were a kind gift of Søren. R. Paludan (Aarhus University) who obtained them from various places. The Wuhan-like early European SARS-CoV-2 B.1 lineage (FR-4286) was kindly provided by Professor Georg Kochs (University of Freiburg), Professor Arvind Patel (University of Glasgow, UK) kindly provided the Alpha variant B.1.1.7, and the Beta variant B.1.351 was kindly provided by Professor Alex Sigal, African Health Research Institute, South Africa. SARS-CoV-2 cluster 5 (Mink variant) (SARS-CoV-2/hu/DK/CL-5/1) and Delta variant B.1.617.2 (SARS-CoV2/hu/DK/SSI-H11) was provided by Statens Serum Institute, SSI, Denmark. B.1 (FR-4286), Alpha, Delta, and cluster-5 variants were propagated using VeroE6-hTMPRSS2, while the beta variant was propagated in human A549-hACE2. In brief, 20 × 10^6^ cells were seeded in different T175 culture flasks and infected the following day at 0.005 MOI in 8 ml of serum-free media (SFM). One hour after infection, culture medium was increased with an extra 10 ml of medium containing serum and virus propagation continued until 72 h post infection. To isolate the virus, cell culture supernatants were removed from the different flasks, centrifuged at 300g for 5 min to remove cell debris, and viruses were concentrated in Amicon filter tubes by spinning down at 4000*g* for 30 min. Concentrated virus was further aliquoted and stored at −80 °C. The amount of infectious virus in the generated stock was determined using a limiting dilution assay (TCID50).

All cell lines used in this study were acquired from ATCC with the exception of the AF2028 and AF2068 lines. Those were a kind gift from Dr Barbara Vanderhyden Ottawa Hospital Research Institute, Canada. Cell lines were cultured either in Dulbecco’s modified Eagle’s medium (DMEM) or RPMI supplemented with 10% fetal bovine serum (FBS).

### Reagents

ESI-09 (α-[(2-(3-Chlorophenyl)hydrazinylidene]-5-(1,1-dimethylethyl)-β-oxo-3-isoxazolepropane-nitrile)), EHT-1864, Rhosin HCl, and ML-141 were purchased from Selleck Chemicals, and CK-666 was purchased from Sigma-Aldrich. All chemicals were >98% pure by HPLC analysis. Inhibitors were prepared as 10 mM stock solutions in DMSO and diluted in SFM for experiments.

### Treatment protocol and reporter assays

Unless noted otherwise, cells were pretreated 2 hours before infection with ESI-09 or other inhibitors in SFM. With the exception of dose-response curves and other analyses where the concentration was explicitly noted, ESI-09 was used at a final concentration of 25 μM. Following the two-hour pretreatment, viruses were added at the indicated MOI in SFM. Cells were incubated for 24 to 48 h, and reporter expression/activity was measured. For VSV, MeV, HSV, and AdV, GFP/mCherry foci were counted using a Cellomics ArrayScan platform. Exposure times and intensity thresholds were optimized for each scan to minimize background in uninfected control wells. For VACV, virus growth was measured *via* Fluc activity using Promega Bright-Glo as a substrate and measuring luminescence with a Biotek Cytation 5 plate reader. In either case, percent inhibition for ESI-09 was calculated by normalizing ESI-09 measurements (F_ESI_) to uninfected (F_min_) and DMSO-treated (F_max_) wells:

$$\%\;Inhibition=100-100\times \frac{F_{ESI}-F_{\min}}{F_{\max}-F_{\min}}$$

### Plaque assays

VSV, VACV, and HSV titers were measured *via* standard plaque assay. Serial dilutions of each virus were prepared in SFM and infected into confluent 12-well plates of Vero (VSV, HSV) or U2OS (VACV) cells. Cells were incubated at 37 °C for 1 (VSV) to 2 (VACV, HSV) hours to allow virus attachment, following which the inoculum was removed and replaced with 1.5 % (VACV, HSV) or 3 % (VSV) carboxymethyl-cellulose overlay. Cells were then incubated for 48 h at 37 °C and then stained with crystal violet. MeV and AdV titers were determined by 50% tissue culture infective dose (TCID50) endpoint method according to Spearman–Karber on Vero (MeV) or HEK293T (AdV) cells (81, 82).

### Viability assays

ESI-09 toxicity was measured using the CyQUANT LDH cytotoxicity assay (Thermo Fisher Sceintific) as per the manufacturer’s guidelines. The assay was performed on HeLa cells treated for 24 h with varying concentrations of ESI-09. A colony formation assay was also performed with Vero cells to measure cell proliferation following ESI-09 treatment. Cells were treated for 24 h with 25 μM ESI-09 in SFM. Cells were then subcultured and seeded into 12-well plates with 500 cells. Cells were incubated for seven days at 37 °C and stained with crystal violet.

### Immunoblotting

Whole cell lysates were prepared in RIPA buffer (Pierce) with protease/phosphatase inhibitors (NEB). Protein concentrations were determined by bicinchoninic acid assay (Pierce). Samples were mixed with NuPage LDS sample buffer (Invitrogen) and loaded into precast SDS-PAGE gels (Bio-Rad). Following SDS-PAGE, proteins were transferred onto nitrocellulose membrane, blocked for 1 h with 5% skim milk in TBS-Tween, and incubated with primary antibody. EPAC1 (5D3), EPAC2 (5B1), ITGβ1 (D2E5), and β-actin (13E5) antibodies were purchased from Cell Signaling Technology and used at a dilution of 1:1000. HRP-conjugated anti-mouse (7076S) and anti-rabbit (7074S) IgG secondary antibodies were purchased from Cell Signaling and used at a dilution of 1:2000. Westerns were developed with Clarity or Clarity Max ECL substrates (Bio-Rad) and imaged using a Bio-Rad GelDoc imaging system.

For experiments involving SARS-CoV-2, immunoblotting was performed as follows; in brief, cells were lysed in 100 ml of ice-cold Pierce RIPA lysis buffer (Thermo Fisher Scientific) supplemented with 10 mM NaF, 1× complete protease cocktail inhibitor (Roche), and 5 IU.mL^−1^ benzonaze (Sigma), respectively. Protein concentration was determined using a bicinchoninic acid protein assay kit (Thermo Fisher Scientific). Whole-cell lysates were denatured for 3 min at 95 °C in the presence of 1× XT Sample Buffer (Bio-Rad) and 1× XT reducing agent (Bio-Rad). Thirty milligrams of reduced samples was separated by SDS-PAGE on 4 to 20% Criterion TGX precast gradient gels (Bio-Rad). Each gel was run initially for 15 min at 70V and 45 min at 120V. Transfer onto polyvinylidene difluoride membranes (Bio-Rad) was done using a Trans-Blot Turbo Transfer system for 7 min. Membranes were blocked for 1h with 5% skim-milk (Sigma Aldrich) at room temperature in PBS supplemented with 0.05% Tween-20 (PBST). Membranes were fractionated in smaller pieces and probed overnight at 4 °C with any of the following specific primary antibodies in PBS Tween 0.05%: anti-spike SARS-CoV-2 (Genetex 1/1000) or anti-Vinculin (Sigma Aldrich 1/10,000) used as loading control. After three washes in PBST, secondary antibodies, peroxidase-conjugated F(ab)2 donkey anti-mouse IgG (H+L) (1:10,000), or peroxidase-conjugated F(ab)2 donkey anti-rabbit IgG (H+L) (1:10,000) (Jackson ImmunoResearch) were added to the membrane in PBST 1% milk for 1h at room temperature. All membranes were washed three times and exposed using either the SuperSignal West Pico PLUS chemiluminescent substrate or the SuperSignal West Femto maximum sensitivity substrate (Thermo Fisher Scientific) and a ChemiDoc Imaging System (Bio-rad).

### siRNA KD

TriFECTa RNAi kits were purchased from IDT for Rapgef3 (EPAC1) and Rapgef4 (EPAC2). TriFECTa kits come with three predesigned DsiRNAs that target different sites in a target gene. In our assays, all three DsiRNAs were combined into a single pool. RNA was prepared as per the manufacturer’s instructions. RNAs were transfected into target cells using the GenMute siRNA transfection reagent (SignaGen). Two days following transfection, cells were infected with VACV or VSV at an MOI of 0.1 and 0.01, respectively. After 24 to 48 h, cells were freeze thawed and virus titers were measured by plaque assay.

### CRISPR/CAS9 KO lines

BxPC3 cancer cells were washed with PBS, trypsinized, and resuspended at 1E5 in 500 μL of SFM in duplicate for KO and Mock KO cell line generation. To prepare 3 μM sgRNA, 1.5 nmol was diluted in 15 μL of nuclease-free TE buffer. To prepare 3 μM Cas9, 20 μM of stock was diluted in nuclease-free water. An initial master mix consisting of 25 μL OptiMEM, 1.3 μL of sgRNA (3 μM) or TE (for Mock KO), 2 μL of Cas9 (3 μM), and 2 μL of Plus reagent was incubated for 10 min at room temperature. A second master mix composed of 25 μL OptiMEM and 3 μL LipoCRISPR max [per cell line] was incubated for 5 min at room temperature. The second master mix was added to the initial master mix and incubated for 10 min. For each cell line, the combined master mix was added to the cells which were divided into two wells. Cells were expanded into 6-well plate format before freezing polyclonal lines or sorting. Monoclonal cell lines were then screened by dot blot for EPAC1 KO.

### Virus entry/binding assays

#### Lentivirus assay

pLenti-CMV Hygro TMPRSS2 and pLenti-CMV Puro ACE2 22 (kind gifts from Dr A.C. Gingras, University of Toronto, Canada) were used to generate lentivirus *via* second generation pSPAX2 and pMD2.G (Addgene) (83). VeroE6 cells (ATCC, CRL-1586) transduced the lentiviruses to generate VeroE6 stably overexpressing hACE2 and hTMPRSS2. VeroE6-ACE2-TMPRSS2 cells were seeded at 2.5E4 cells/well in 96-well dishes. Spike pseudotyped lentiviruses were generated as previously described (83). The next day, the cells were infected with Spike pseudotyped lentivirus encoding firefly luciferase reporter and treated with ESI-09. Sixteen hours post infection, Bright-Glo Luciferase assay system (Promega) was added to the samples and luminescence was measured with a Biotek Cytation 5 plate reader.

A549 cells were pretreated with ESI-09 or DMSO for 2 h and infected with the replication-deficient AdV-ΔE1 virus. After 24 h, mCherry-positive cells were counted and the percent inhibition was calculated as described above.

Virus attachment/entry to cells pretreated with 25 μM ESI-09 was measured by incubating each of the viruses at an MOI of 20 with Vero (VSV, MeV, HSV) or U2OS (VACV) cells for 1 to 2 h at 37 °C. Cells were then washed three times with ice cold PBS, scraped from the plate, and freeze-thawed to release virus. The freeze-thawed mixture was then titered by plaque assay to determine the number of virus particle that were bound to the cells.

### Immunofluorescence staining of VACV and VSV entry

U2OS (for VACV) or Vero (for VSV) cells were seeded onto glass coverslips in 6-well plates. Once cells had adhered, they were treated with ESI-09 or other inhibitors for 2 h before infecting with VACV or VSV at an MOI of 10. Cells were then incubated for around 2 h at 37 °C to allow virus attachment and entry. Cells were then fixed with 4% paraformaldehyde and in some instances permeabilized with 0.2% Triton X-100. Cells were then blocked with 1% bovine serum albumin for 30 min before staining with either a polyclonal VACV virus (1:1000; LS-C103289; LSBio) or VSV (1:1000, prepared in house *via* immunization of rabbits (84)) antibody. VACV-infected cells were then washed and stained with an Alexa Flour 594 anti-rabbit IgG secondary (A11037, Invitrogen), while VSV was stained with an Alexa Flour 488 anti-rabbit IgG secondary (A21206, Invitrogen). Coverslips were then mounted using prolong with DAPI (comp) and imaged with a Zeiss Axioskop 2 Epi-Fluorescence Microscope.

### Actin staining

Following seeding, cells were treated and infected using the standard protocol described above. After 24 h, cells were fixed with 4% paraformaldehyde and stained with Phalloidin-iFluor 647 Reagent (Abcam) following the manufacturer’s protocol. Coverslips were then mounted using prolong with DAPI (comp) and imaged with a Zeiss Axio Imager M1 microscope.

### Syncytia colocalization assay

Stable cell lines of HeLa cells expressing mCherry or eGFP were generated *via* lentiviral transduction. For colocalization assay, the mCherry and eGFP cells were mixed together equally and seeded into 12-well plates. Once the cells had reached 80 to 90% confluency, they were transfected with a pcDNA3.1 plasmid containing the P14 fusogen gene using lipofectamine 2000. The transfected cells were incubated for 4 hours, and then the transfection mix was removed and replaced with the inhibitors in SFM. All inhibitors except CK-666 were used at a concentration of 25 μM. CK-666 was used at a concentration of 100 μM. The cells were incubated overnight at 37 °C and then imaged with an EVOS M5000 imagine system. The colocalization analysis was performed with the Celleste 5 software.

### SARS-CoV-2 experiments

VeroE6-TMPRSS2 cells, A549 cells expressing the human ACE2 receptor were seeded at 1.5 × 10^5^ cells per well in complete DMEM in a 24-well plate, whilst Calu3 cells were seeded at 3 x 10^5^ cells per well. The following day, cells were pretreated with ESI-09 (50 μM) for 2 h, prior to infection. SARS-CoV-2 (either as the original Freiburg strain or the different VOCs) was added as a MOI of 0.1 for 1 h, in the presence of the drug. After 1 h, the virus containing medium was removed and replaced with fresh complete DMEM containing 50 μM of ESI-09, for 48 h. Cells were then rinsed in PBS and lysed in 400 μl of lysis buffer (Roche, 11828665001) followed by RNA extraction and gene expression analysis. For the experiments investigating the mode of action of ESI-09, the drug treatment was added full time, as described above, during pretreatment only (entry) or only postinfection (post entry).

For experiments involving native SARS-CoV-2, gene expression was determined by real-time quantitative PCR, using TaqMan detection systems (Applied Biosciences). RNA was extracted using the High Pure RNA Isolation kit (Roche, 11828665001) according to the manufacturer’s instructions with RNA being eluted in 60 μl of sterile RNAse-free water. RNA quality and purity were further assessed by Nanodrop spectrometry (Denovix DS-11). RNA levels were analyzed using premade TaqMan assays and the RNA-to-Ct-1-Step kit according to the manufacturer’s recommendations (Applied Biosciences). We used the commercially available Taqman assay (Thermo Fisher Scientific, cat. no. 4392938), and samples were analyzed in a 10 μl (final volume) reaction mix containing 5 μl Master Mix, 0.2 μl RT Enzyme, 3.64 μl Nuclease-free water, 0.16 μl of primers (TBP, ACTB, Thermo Fisher Scientific), and 1 μl of either pure or diluted RNA where mentioned. For the SARS-CoV-2 gene, primers and probe sequences were provided by the CDC and purchased from Eurofins. Samples were analyzed in a final volume of 10 μl, containing 5 μl of Master Mix, 0.5 μl fw primer (10 pmol/μl fw primer - AAATTTTGGGGACCAGGAAC), 0.7 μl rev primer (10 pmol/μl rev primer – TGGCACCTGTGTAGGTCAAC), × 0.2 μl probe (20 pmol/μl – FAM-ATGTCGCGCATTGGCATGGA-BHQ), × 2.4 μl Nuclease-free water, and 1 μl of RNA. Data analysis was performed on a Applied Biosciences qPCR platform with program: 2′50 °C; 2′95 °C; 40×(1”95 °C; 20”60 °C). Ct values were extracted using the Applied Biosystems Software (https://www.thermofisher.com/ca/en/home/technical-resources/software-downloads/ab-secondary-data-analysis-modules/applied-biosystems-analysis-modules-for-qpcr.html).

### SARS-CoV-2 immunofluorescence and confocal microscopy

Calu3 cells were seeded onto glass coverslips placed on the bottom of 12-well plates and treated with either DMSO (0.1 %) or ESI-09 (50 μM) for 2 h prior challenge with SARS-CoV-2 (MOI of 0.1). After 48 h, cells were washed with Dulbecco's phosphate-buffered saline (DPBS) and fixed for 40 min in 4 % paraformaldehyde following a 20-min permeabilization with 0.2% Triton X-100 in DPBS. Next, blocking with 2 % FBS in DPBS was performed for 40 min, and mouse SARS-CoV-2 spike antibody (1:200, GeneTex) was applied for 1 h at room temperature in blocking solution. After three washes with DPBS, cells were incubated with goat anti-mouse Alexa Fluor 555 nm fluorophore-conjugated secondary antibody (1:400, Invitrogen), Alexa Fluor Plus 647 Phalloidin (1:400, Invitrogen), and PureBlu DAPI nuclear staining (1:100, Bio-Rad) for 1 h at room temperature in the dark. Cells were then washed three times with DPBS and mounted onto microscope slides using ProLong Gold Antifade mounting medium (Invitrogen). Slides were air-dried in the dark and examined on the next day using a Zeiss LSM 710 Inverted Confocal Microscope with corresponding Zeiss Zen software (https://www.zeiss.com/microscopy/en/products/software/zeiss-zen.html).

### Murine explants

Six-week old BALB/c mice were ordered from Charles River Laboratories. Lungs and ovaries were removed and stored in RPMI media containing Pen-Strep. Lungs were processed into 2-mm thick sections and cored using 2 mm Integra biopsy punches (Thermo Fisher Scientific, Cat No 12-460-399). Lung cores were then placed in 24-well plates with DMEM media containing 10% FBS, 30 mM Hepes, Pen-Strep (Gibco), 75 μg/ml gentamicin, and 0.25 mg/ml amphotericin B. Unprocessed ovaries were also transferred into 12-well plates containing 1 ml of antibiotic-containing DMEM. Tissues were pretreated with 0.01 % DMSO or 25 μM ESI-09 for 1 h before infecting with 1E6 pfu of VACV (expressing Fluc and GFP). Three days after infection, tissue viability was measured by Alamar assay, cores were homogenized by vigorous agitation with metal beads, and virus titers were measured by plaque assay.

### Human patient explants

Collection of human tissue for this study was approved by the Global Tissue Consent and Collection Program at the Ottawa Hospital Research Institute. Tumors were surgically removed from two patients: a 63 year old male with kidney carcinoma (GTC322) and a 47 year old female with ovarian/endometrial adenocarcinoma (GTC340). Tumors were sectioned into 2 mm layers and cored using 2 mm Integra biopsy punches (Thermo Fisher Scientific, Cat No 12-460-399). Tumor cores were placed in 24-well plates with DMEM mediate containing 10% FBS, 30 mM Hepes, Pen-Strep (Gibco), 75 μg/ml gentamicin, and 0.25 mg/ml amphotericin B. The viability of each core was then measured by Alamar assay after incubating with resazurin dye overnight. In the morning, the media was replaced and the cores were infected with 1E5 PFU of SKV viruses. Virus replication in the tumor cores were monitored by virus-expressed eGFP, and once infection reached levels of around 50%, another overnight Alamar assay was performed to measure changes in viability. The following day, cores were collected, homogenized by agitation with metal beads, and titered by plaque assay.

### *In vivo* challenge experiments

All mouse experiments were approved by the University of Ottawa institutional review board for animal care. Six-week old CD1 nude mice were ordered from Charles River Laboratories. The Tiantan strain of VACV was used for challenge experiments consistent with the approaches outlined previously (33). This virus expressed Firefly luciferase from a constitutive early/late promoter. For I.N. model, mice where anesthetized with isofluorane and administered 1E6 pfu of VACV I.N. ESI-09 or vehicle solutions was administered at a dose of 36 μg/kg/day for five days starting on the same day they were injected with the virus. The inhibitor was prepared in PBS with 5% DMSO. Weight change was tracked to monitor disease progression, and IVIS was run four days after infection to track virus replication. Mice were endpointed once weight loss exceeded 20% or when they began developing severe pox lesions. *In vivo* imaging was performed as described previously (33).

### Statistical tests

All data was processed and analyzed in GraphPad Prism. Statistical significance was determined using 2-way ANOVA Fisher’s LSD test (∗, *p* < 0.0361; ∗∗, *p* < 0.0021; ∗∗∗, *p* < 0.0002; ∗∗∗∗, *p* < 0.0001).

## Data availability

All relevant data can be found in main text and supporting information. Raw data is available upon request by contacting Stephen Boulton at sboulton@ohri.ca.

## Supporting information

This article contains supporting information.

## Conflicts of interest

R. S. is currently employed by the Public Health Agency of Canada (PHAC). His contribution to the study described herein was conducted prior to his employment at PHAC. The work was not undertaken under the auspices of PHAC as part of their employment responsibilities and does not represent the views of PHAC. All other authors have declared that they have no conflicts of interest with this study.
