# Supplementary material for: Inhibition of exchange proteins directly activated by cAMP as a strategy for broad-spectrum antiviral development
Source: J Biol Chem. 2023 Apr 24;299(6):104749. doi: 10.1016/j.jbc.2023.104749 (PMC10124099; doi:10.1016/j.jbc.2023.104749)
Supplement: Supporting information [file mmc1.docx]

**Supporting Information for:**

**Inhibition of Exchange Proteins Directly Activated by cAMP as a Strategy for Broad-Spectrum Antiviral Development**

Stephen Boulton^1,2,*^, Mathieu J.F. Crupi^1,2^, Siddharth Singh^1,2^, Madalina E. Carter-Timofte^4^, Taha Azad^1,2^, Bailey C. Organ^1,2^, Xiaohong He^1,2^, Rida Gill^1,2^, Serge Neault^1,2^, Taylor Jamieson^1,2^, Jaahnavi Dave^1,2^, Naziia Kurmasheva^4^, Bradley Austin^1^, Julia Petryk^1^, Ragunath Singaravelu^1,2,5^, Ben Zhen Huang^1,2^_,_ Noah Franco^1,2^, Kaaviya Babu^1,2^, Robin J. Parks^1,2,3^, Carolina S. Ilkow^1,2^, David Olagnier^4^, John C. Bell^1,2,3,^*

# Affiliations:

1Cancer Therapeutics Program, Ottawa Hospital Research Institute, Ottawa, ON, K1H 8L6, Canada

^2^Department of Biochemistry, Microbiology and Immunology, University of Ottawa, Ottawa, ON, K1H 8M5, Canada

^3^Department of Medicine, University of Ottawa, Ottawa, ON, K1H 8M5, Canada

^4^Aarhus University, Department of Biomedicine, Aarhus C, 8000, Denmark

^5^Faculty of Medicine and Health Sciences, Department of microbiology and infectious diseases, Université de Sherbrooke, Sherbrooke, QC J1E 4K8, Canada

^6^Centre de Recherche du CHUS, Sherbrooke, QC J1H 5N4, Canada

^7^Public Health Agency of Canada, Ottawa, Ontario, Canada, K1A 0K9

*Correspondence to Stephen Boulton, [sboulton@ohri.ca](mailto:sboulton@ohri.ca) or John C. Bell, [jbell@ohri.ca](mailto:jbell@ohri.ca)

**Supporting Figures:**


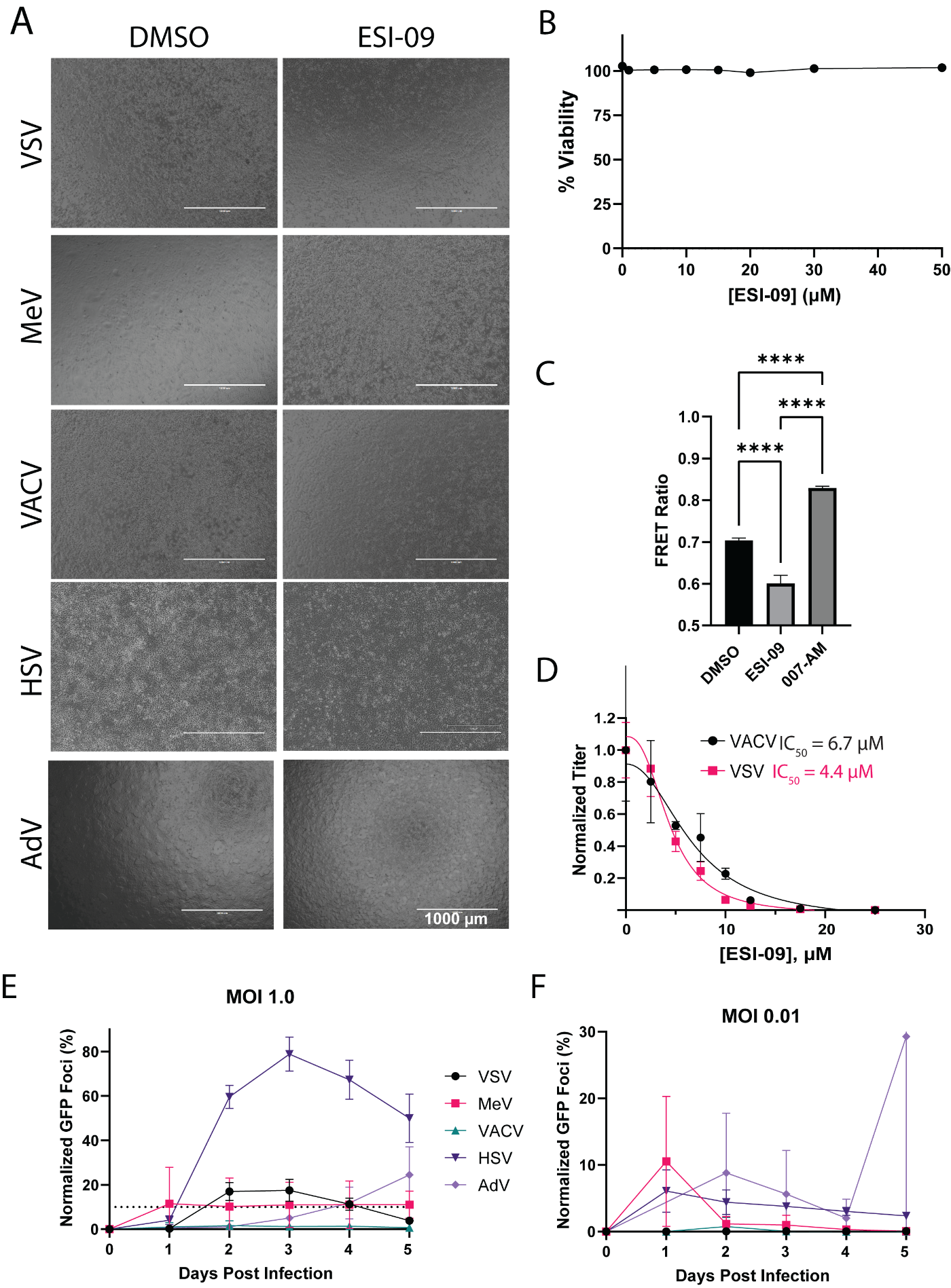


**Figure S1:** *Optimization of ESI-09 dose and treatment regimens*. **A)** Brightfield images of cells from Fig. 1A. **B)** Cell viability from LDH release assay 24 hours after treatment with ESI-09 (n=3). **C)** Rap1 FRET biosensor assay in HEK293T cells treated with ESI-09 or 007-AM. The FRET ratio is correlated with the interaction between Rap1 and the Rap-binding domain of its downstream target RalGDS. **D)** Dose-response inhibition of VACV and VSV with 25 µM ESI-09 in U2OS and Vero cells, respectively. Cells were pretreated with ESI-09 for 2 hours prior to infection with VACV at an MOI of 0.1 and VSV at an MOI of 0.01. **E-F)** Kinetic response of ESI-09 treatment with different viruses at MOIs of 1.0 and 0.01. Statistical significance determined by Fisher’s LSD test (*, P < 0.0361; **, P < 0.0021; ***, P < 0.0002; ****, P < 0.0001)


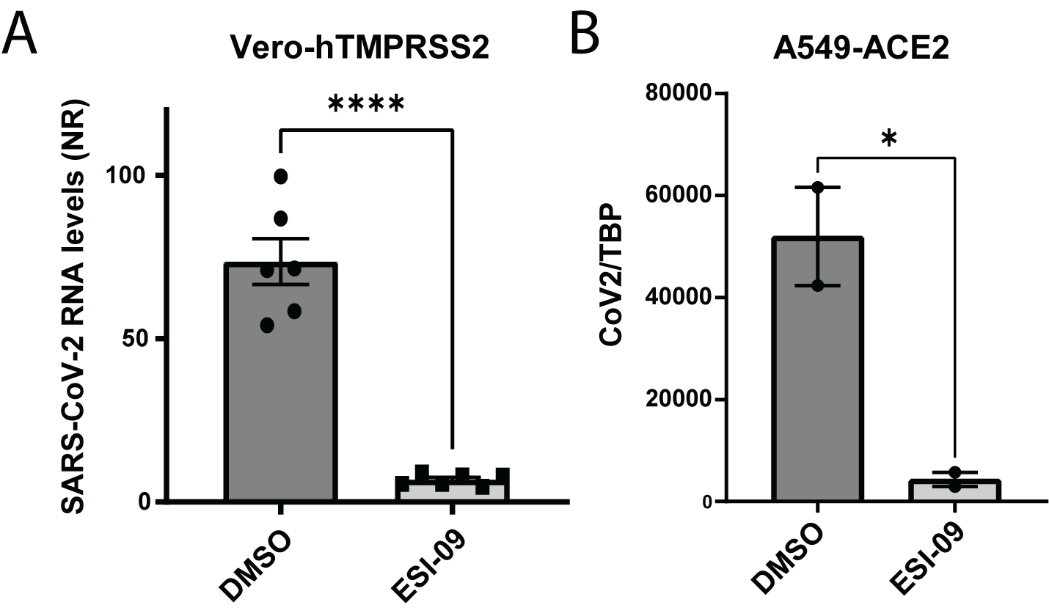


**Figure S2:** *Anti-viral activity of ESI-09 against the original SARS-CoV-2 and variants of concern*. **A - B)** Vero E6 cells expressing hTMPRSS2 and A549 cells expressing ACE2 were pre-treated with 50 µM of ESI-09 for 2h, followed by infection with SARS-CoV-2 (MOI 0.1) for 48h. SARS-CoV-2 RNA levels were quantified by RT-qPCR. Statistical significance determined by Fisher’s LSD test (*, P < 0.0361; **, P < 0.0021; ***, P < 0.0002; ****, P < 0.0001)


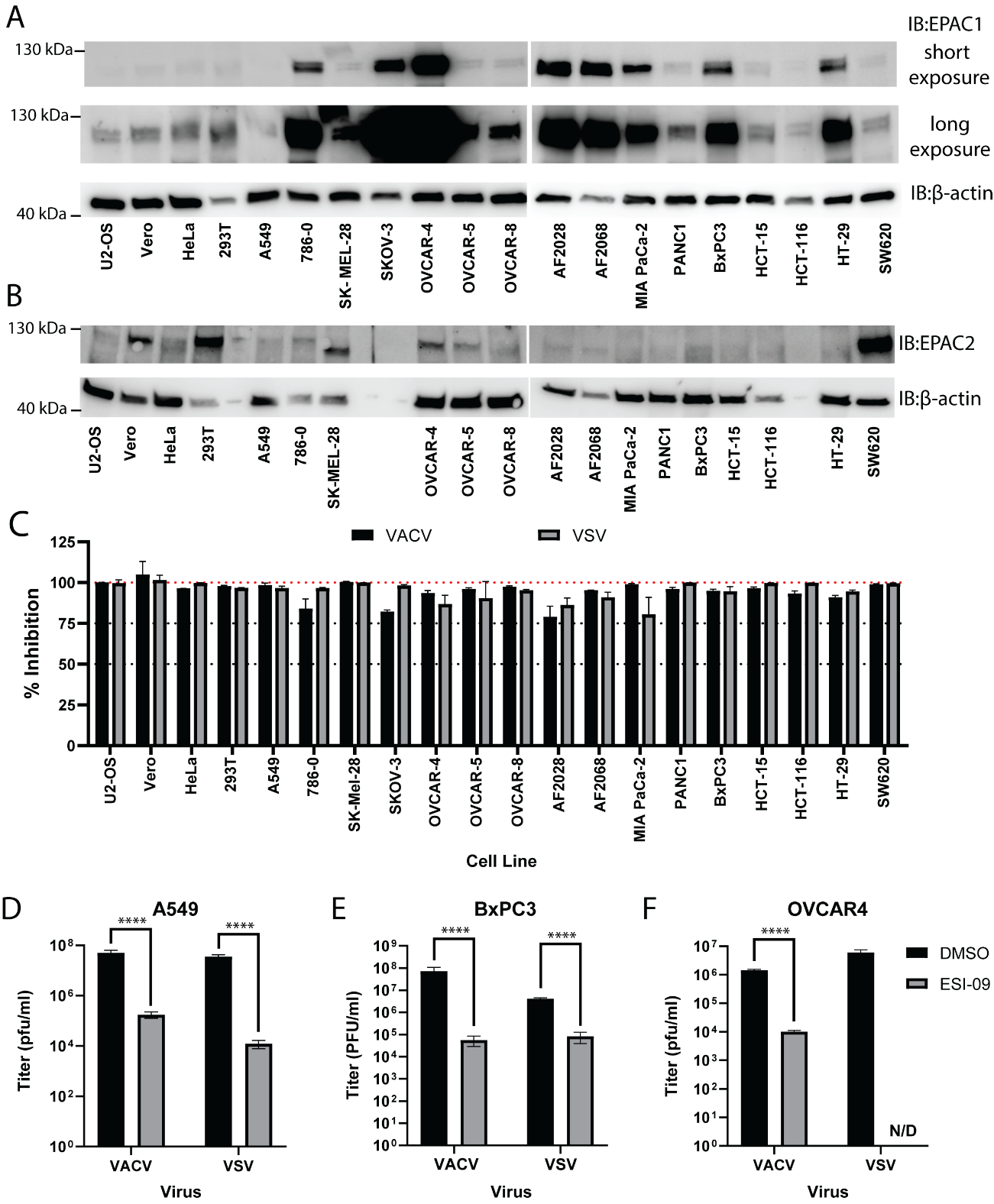


**Figure S3:** *Inhibition of VACV and VSV by ESI-09 in different cell lines*. **A)** Western blot of cell line panel with EPAC1 antibody. The top images were taken with a short (1 sec) exposure time while the middle panel was acquired with a long (30 sec) exposure time. **B)** EPAC2 western blot performed similarly to panel A. Images were acquired with 120 sec (left) and 20 sec (right) exposure times **C)** ESI-09 mediated inhibition of VACV or VSV replication in cell line panel. Percent inhibition was measured according to reporter signal relative to DMSO control. The MOI for VACV and VSV was 0.2 and 0.05, respectively. **D-F)** Virus titers for VACV and VSV in cell lines with low (A549), medium (BxPC3) and high (OVCAR-4) levels of EPAC1 expression. Statistical significance determined by Fisher’s LSD test (*, P < 0.0361; **, P < 0.0021; ***, P < 0.0002; ****, P < 0.0001)


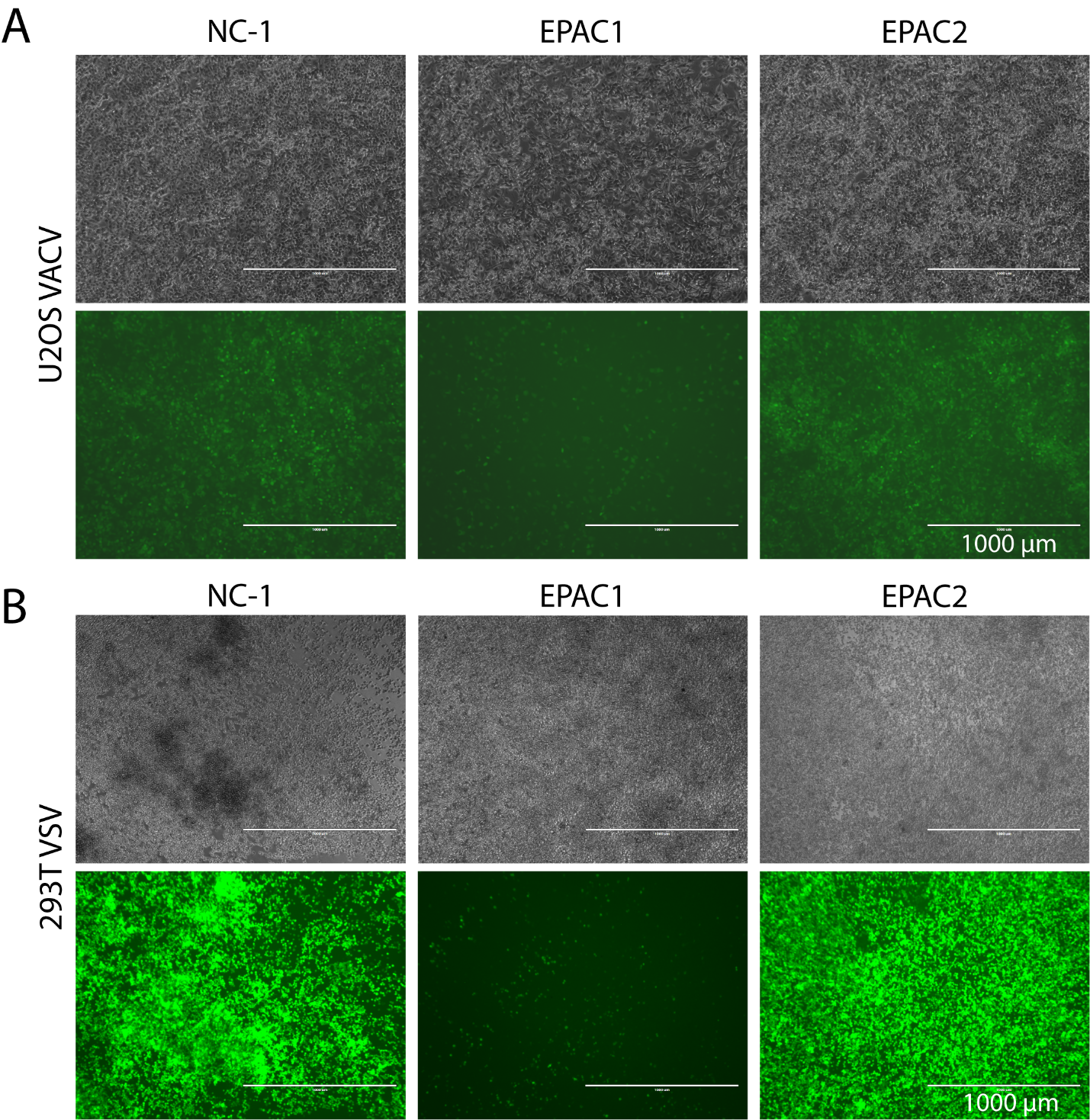


**Figure S4:** E*SI-09 blocks VACV and VSV infection through inhibition of the EPAC1 isoform*. **A)** BF and GFP images from cells infected with vaccinia at an MOI of 0.1 after selective knockdown of EPAC1 or EPAC2 with siRNA. NC-1 is scrambled RNA. **B)** Similar to panel C except with VSV in 293T cells.


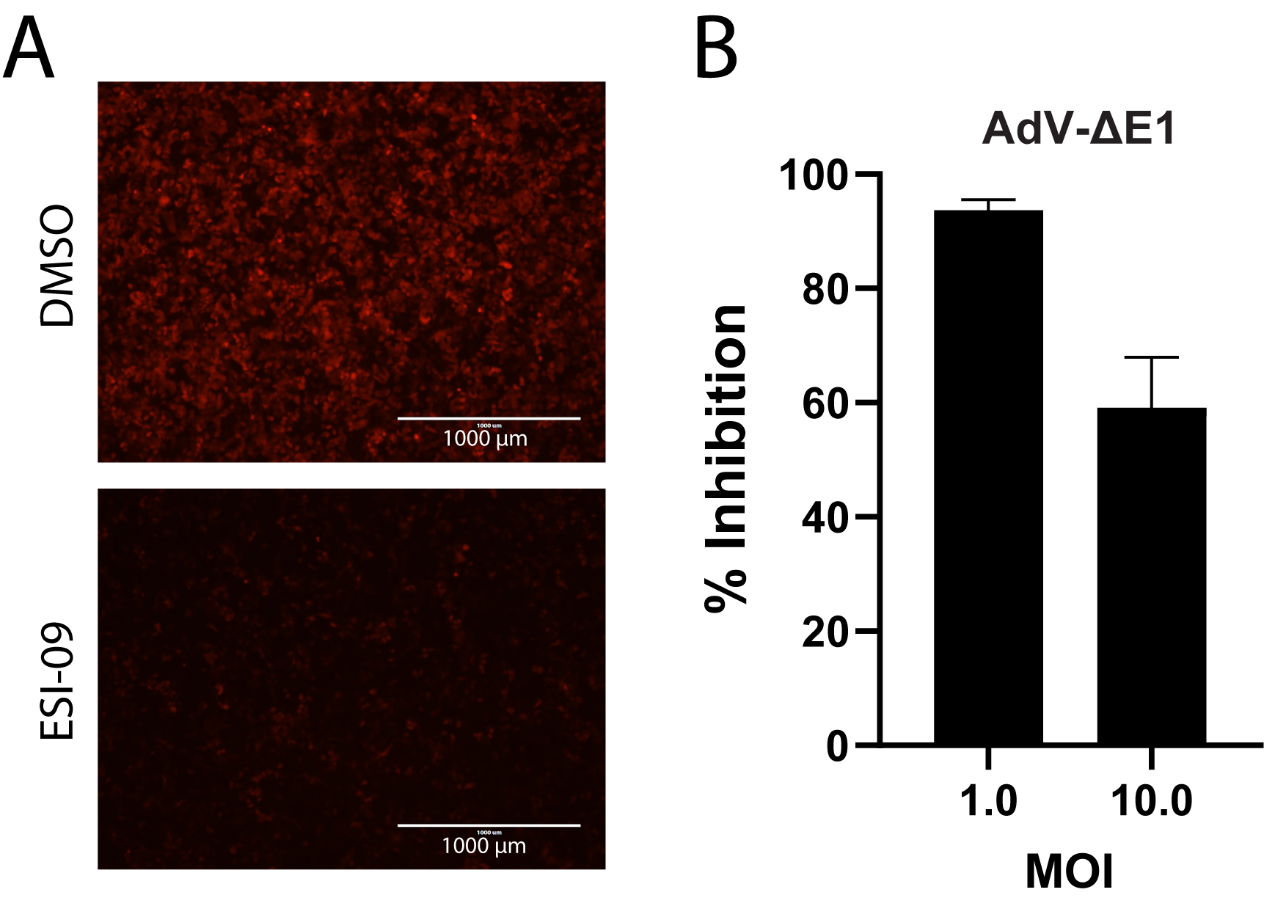


**Figure S5:** ESI-09 inhibits replication deficient E1-deleted AdV. **A)** Images of A549 cells treated with 25 µM ESI-09 or DMSO and infected with AdV-ΔE1 at an MOI of 10. **B)** Percent inhibition of AdV-ΔE1 in cells treated with ESI-09.


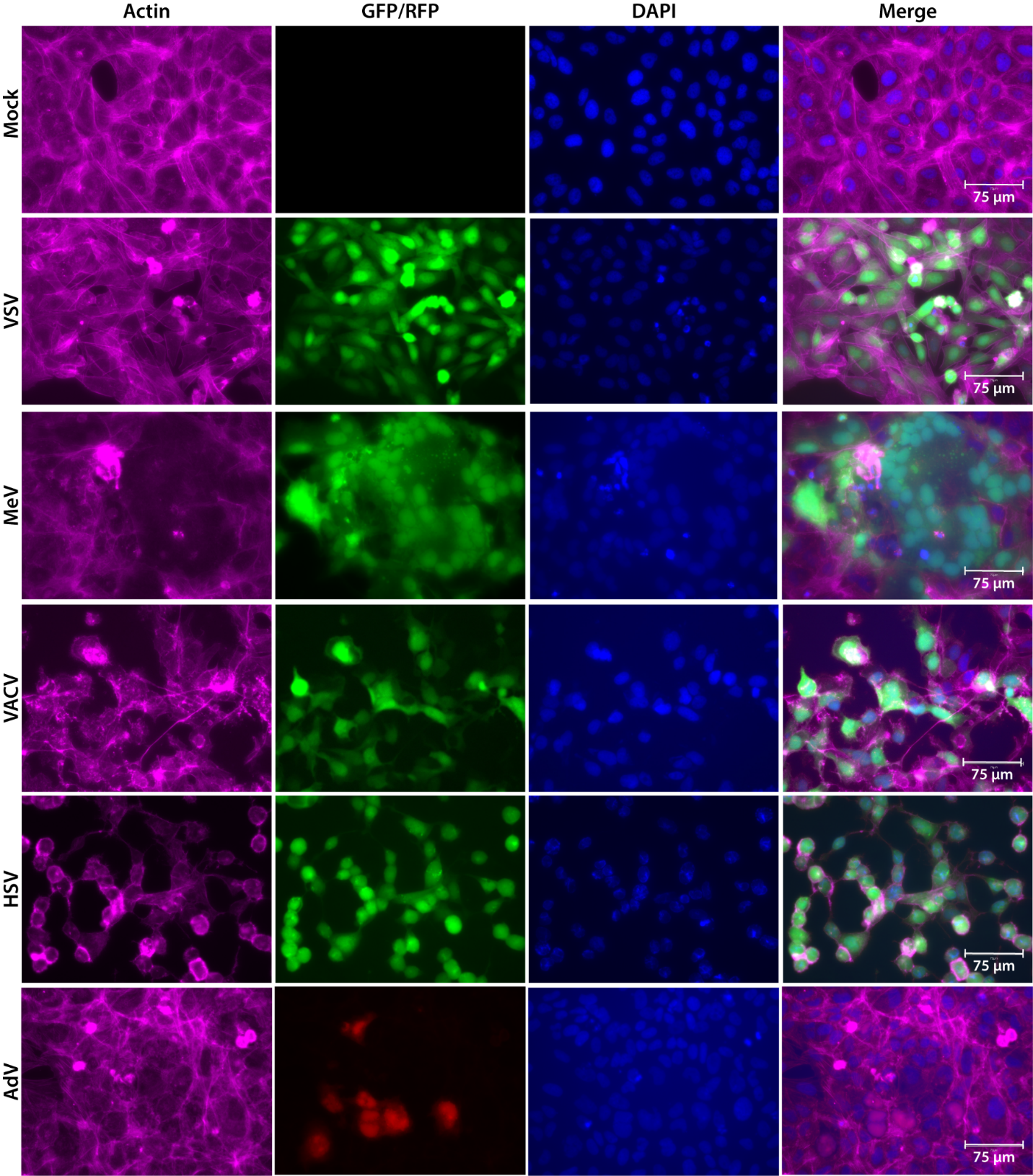


**Figure S6:** *Actin undergoes remodeling in virally infected cells*. **A)** Phalloidin staining of actin filaments (magenta) in U2OS cells infected with VSV, MeV, VACV, HSV and AdV. Samples were stained 24 hours after infection. GFP/RFP were expressed endogenously from the viruses.


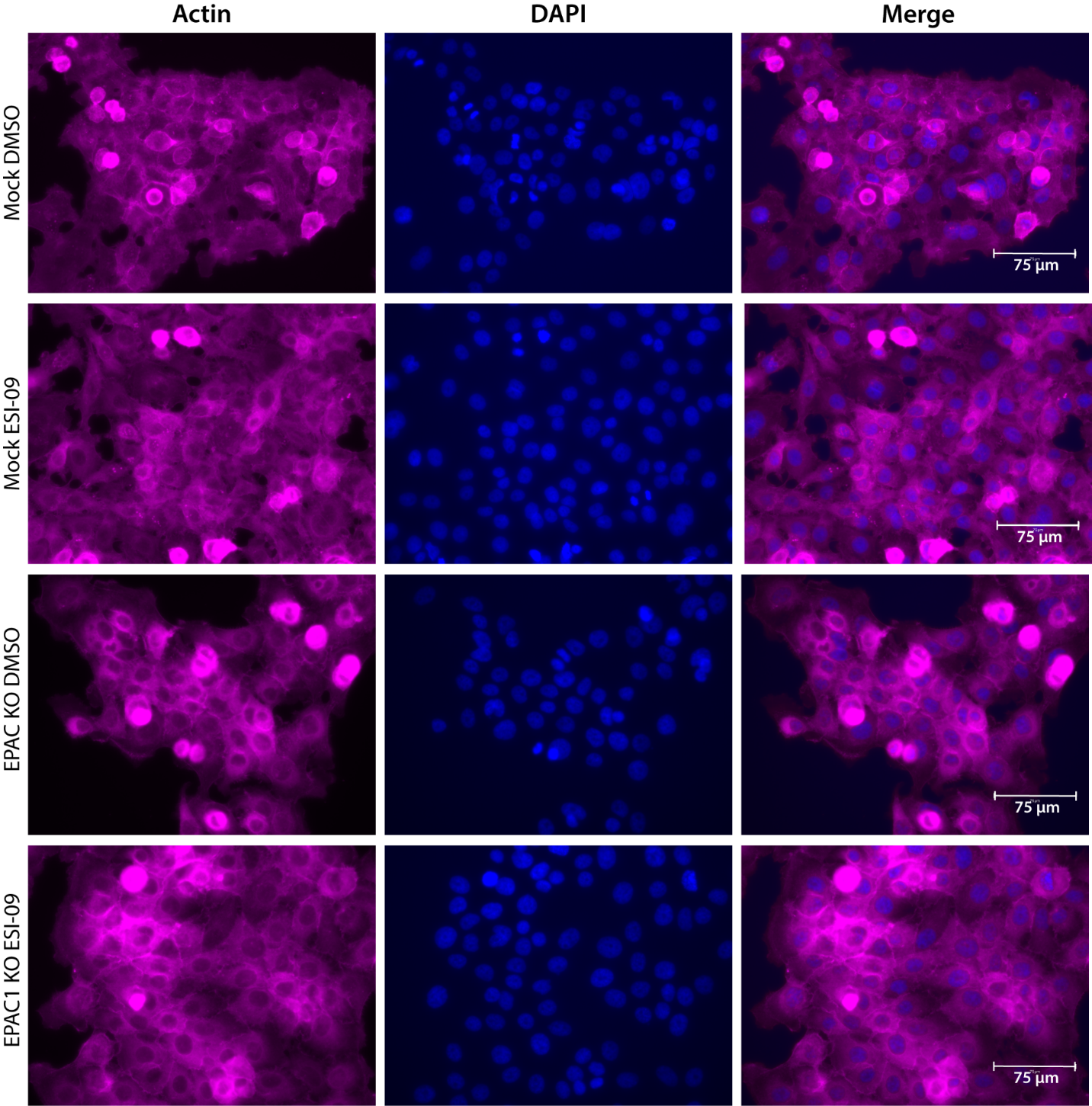


**Figure S7:** *Actin remodeling in EPAC1 Knockout BxPC3 cells*. Native and BxPC3 with EPAC1 knocked out were treated with 25 µM ESI-09 or vehicle and stained with phalloiden-AF647 to examine changes in actin morphology.


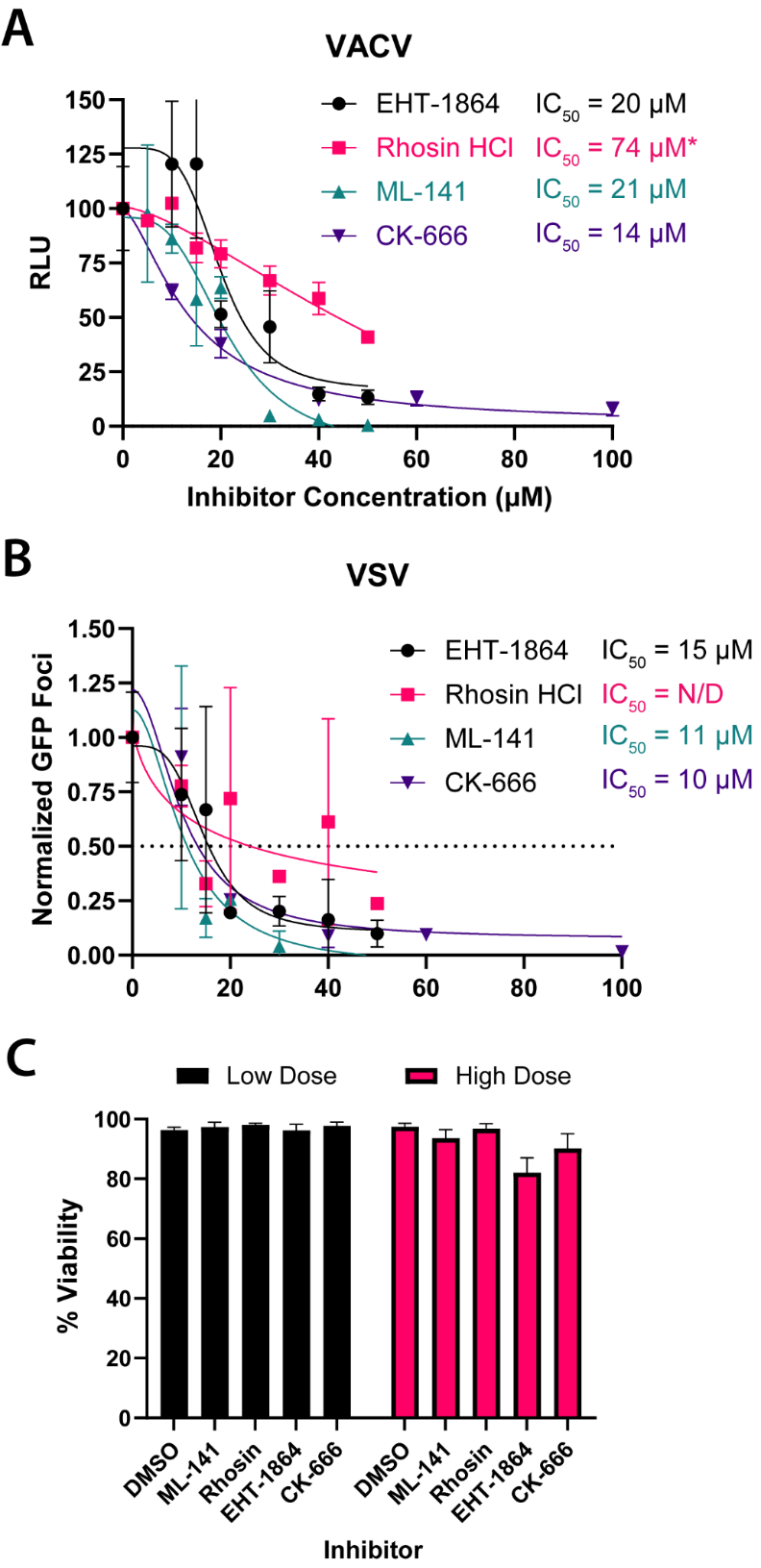


**Figure S8:** *Characterization of GTPase inhibitors*. **A-B)** Dose-response curves for GTPase inhibitors against VACV or VSV. The IC50 for Rhosin against VACV was determined without a complete confidence interval (marked by asterisk). **C)** Cell viability in cells treated for 24 hours with GTPase inhibitors, measured by LDH release assay. The low dose is 12.5 µM for all inhibitors except CK-666, which was 50 µM. The high dose was double the concentration of the low dose.


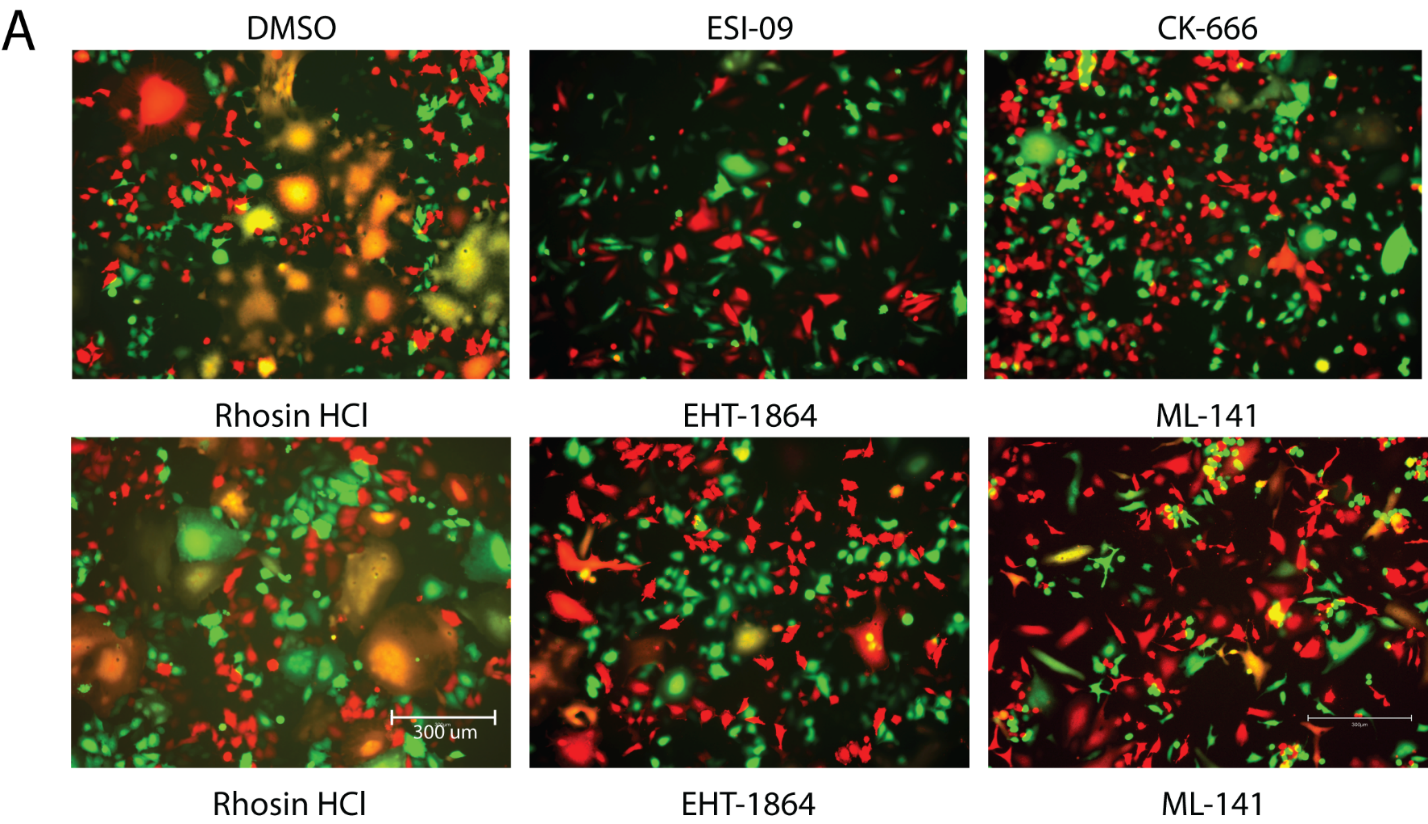


**Figure S9:** *Effect of GTPase inhibitors on P14-induced syncytia*. **A)** HeLa cells expressing either eGFP or mCherry were combined in equal amounts and transfected with the P14 construct. After four hours, the cells were treated with the GTPase inhibitors at a concentration of 25 µM, except CK-666, which was treated with 100 µM. Images were then taken after 24 hours. A magnified image of the same sample from Figure 4D was used for the DMSO control to allow a direct comparison here of syncytia formation in non-treated cells.


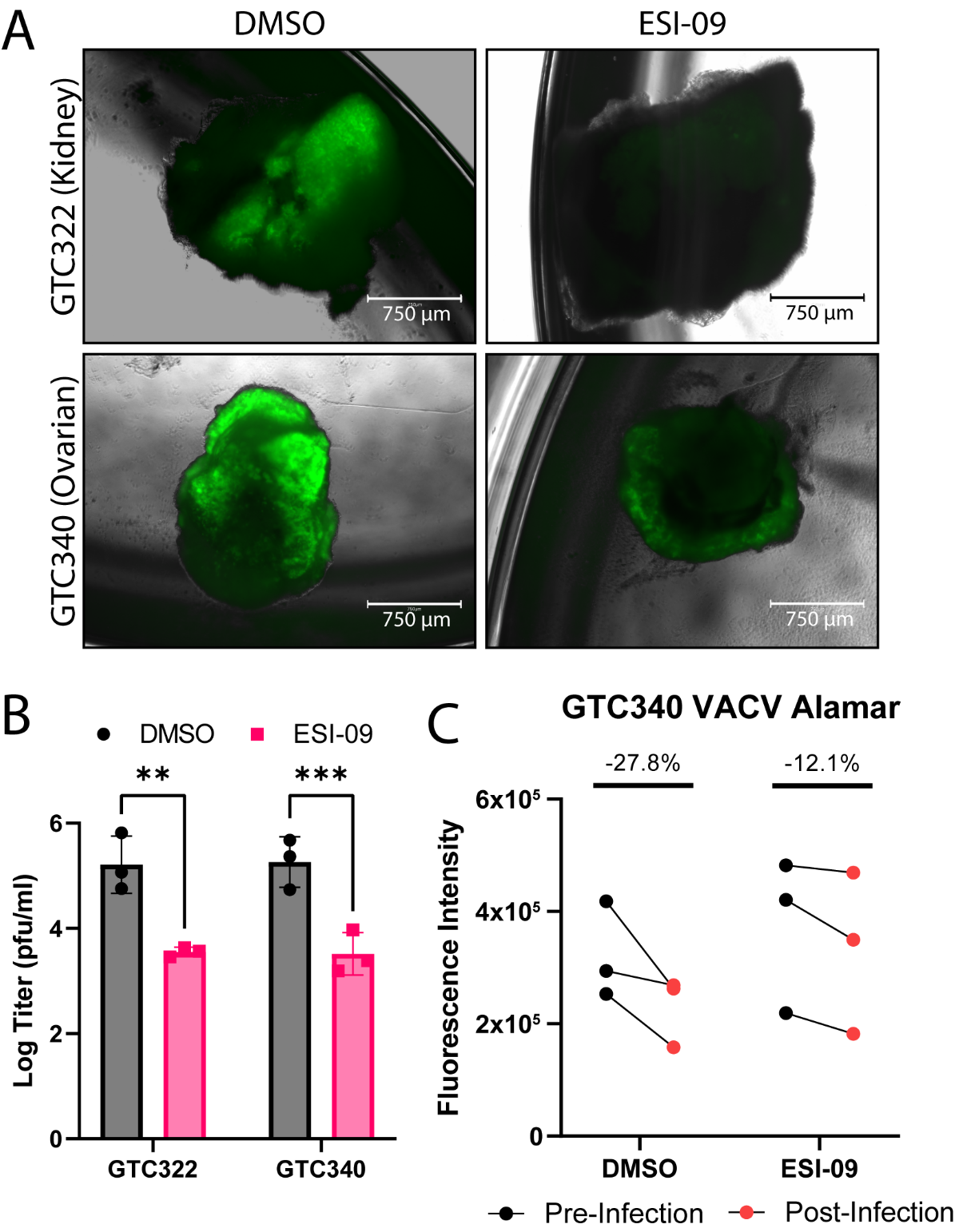


**Figure S10:** *ESI-09 prevents VACV infection in human tissue samples*. **A)** Kidney and ovarian tumors from human patients were processed into 2mm cores and infected with VACV with and without ESI-09 treatment. Fluorescent images were acquired 2 days after infection to monitor virus replication via virus-expressed GFP. **B)** Plaque assay results from infected tissues from panel A. **C)** Changes in tumor core viability following VACV infection. The value at the top indicates the average percent decrease in viability of the tumor cores. Statistical significance determined by Fisher’s LSD test (*, P < 0.0361; **, P < 0.0021; ***, P < 0.0002; ****, P < 0.0001)


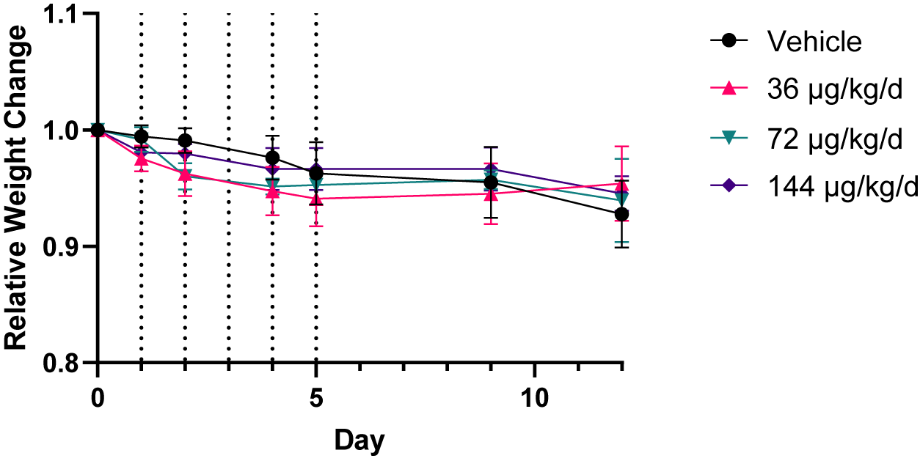


**Figure S11:** *ESI-09 toxicity in mice via IN administration*. 6-week old nude mice were administered ESI-09 at the indicated doses for 5 consecutive days and weights were measured to monitor signs of toxicity.
